# Influence of Blueberry Mosaic Disease on Polyphenolic Profile and Antioxidant Capacity of Highbush Blueberry ‘Duke’ Fruits

**DOI:** 10.3390/antiox14111302

**Published:** 2025-10-29

**Authors:** Nemanja Miletić, Danijel D. Milinčić, Mirjana B. Pešić, Biljana Lončar, Marko Petković, Bojana Vasilijević, Darko Jevremović

**Affiliations:** 1 Food Technology Department, Faculty of Agronomy, University of Kragujevac, Cara Dušana 34, 32102 Čačak, Serbia; marko.petkovic@kg.ac.rs; 2 Department for Chemistry and Biochemistry, Faculty of Agriculture, University of Belgrade, Nemanjina 6, 11080 Belgrade, Serbia; danijel.milincic@agrif.bg.ac.rs (D.D.M.); mpesic@agrif.bg.ac.rs (M.B.P.); 3 Faculty of Technology Novi Sad, University of Novi Sad, Bulevar cara Lazara 1, 21000 Novi Sad, Serbia; cbiljana@uns.ac.rs; 4 Department for Fruit Protection and Certification of Planting Material, Fruit Research Institute, Kralja Petra I 9, 32102 Čačak, Serbia; bvasilijevic@institut-cacak.org (B.V.); djevremovic@institut-cacak.org (D.J.)

**Keywords:** highbush blueberry (*Vaccinium corymbosum* L.), blueberry mosaic associated ophiovirus, mosaic disease, PCR, UHPLC Q-ToF MS

## Abstract

Blueberry mosaic virus (BlMaV) is a persistent pathogen that alters host physiology; however, its impact on secondary metabolism in blueberry fruits remains poorly characterized. In this study, the phenolic profile of the cultivar ‘Duke’ was systematically examined in healthy and BlMaV-infected plants over two successive years. Using UHPLC Q-ToF MS, a total of 46 phenolic compounds were detected, spanning flavonols, phenolic acids, and anthocyanins. Comparative analyses revealed consistent shifts in metabolite abundance between healthy and infected samples. Several flavonol aglycones and phenolic acid derivatives accumulated in infected fruits, whereas multiple anthocyanins and glycosides were reduced. To further explore metabolic relationships, color correlation analysis highlighted distinct co-variation patterns among compound classes. Principal component analysis clearly separated infected and healthy fruits, confirming that viral infection was the dominant source of variation, surpassing the influence of harvest year or environmental factors. Nevertheless, the antioxidant capacity remained unchanged, regardless of the presence of the virus or the variation in environmental conditions. These results provide novel biochemical evidence that BlMaV infection reshapes the phenolic composition of blueberries and lays the groundwork for future studies on the metabolic consequences of viral stress in fruit crops.

## 1. Introduction

Highbush blueberry (*Vaccinium corymbosum* L.) is an appreciated berry fruit whose cultivation is rapidly growing around the world. The popularity of blueberries among consumers increased with new information on their nutritional benefits [1]. The world production of blueberries is estimated at 1.2 million tons in 2023 [2], with the United States as the largest producer. Over the last two decades, blueberry plantations in Serbia expanded rapidly, reaching more than 3000 hectares today [3]. Plantations are highly intensive, with blueberries grown in the soil or substrate in pots, under anti-hail nets, and managed according to integrated or organic farming principles. Blueberries are becoming an important export commodity to diverse markets. Blueberry ‘Duke’ is one of the most well-known and economically important cultivars throughout the world. According to estimations, ‘Duke’ occupies about 80% of the orchards in Serbia. It was bred and released in the United States in 1986 [4]. ‘Duke’ is known for early ripening, high yields, and long shelf life. Fruits are medium-to-large-sized, uniform, firm, light blue, mild, and sweet.

Highbush blueberry is a host to more than 15 viruses and virus-like agents [5]. Ophiovirus vaccinii (blueberry mosaic-associated ophiovirus, BlMaV) has been recently characterized [6], even though the disease it causes has been known for decades [7]. Typical leaf symptoms include white, yellow, light green and pink mosaic and patterns. The symptoms may be localized on the leaves of one or two stems or cover the entire bush. Latent BlMaV infections are reported, and depending on the year, symptoms may be latent for some period and then reappear the following year. The symptoms of blueberry mosaic disease were described in many countries, but BlMaV was officially confirmed in the USA, Serbia, Slovenia, Turkey, Japan, Poland, and Germany [8].

Information on the influence of BlMaV on blueberries is scarce. No data on its economic importance is available. The influence of BlMaV on pomological properties (fruit length, width and height, shape, fruit weight, and soluble solids content) of blueberry ‘Duke’ fruits was reported by Jevremović et al. [9]. The results confirmed no statistically significant impact of BlMaV on the fruit traits examined. The aim of our study was to evaluate the influence of blueberry mosaic disease caused by blueberry mosaic-associated ophiovirus on polyphenolic profile and antioxidant capacity of blueberry ‘Duke’ fruits. For this purpose, a detailed UHPLC Q-ToF MS profiling and identification of all phenolic compounds derived from fruits of healthy and infected blueberry plants was conducted, in order to clarify a potential connection between the accumulation of phenolic compounds and the secondary metabolism of the analyzed blueberry plants.

## 2. Materials and Methods

### 2.1. Leaf and Fruit Sampling

The material for this study was collected in 2019 and 2020 from a blueberry orchard located in Budoželja, Ivanjica (43°32.262′ N 20°16.568′ E). The orchard was located at an altitude of 652 m on a parcel with a gentle slope. Blueberries were grown directly in the soil at 2.5 × 1.5 m spacing, following the principles of integrated pest and disease management system. For the study, we randomly selected two groups of 20 plants from the orchard. The first group included 10 plants exhibiting clear mosaic symptoms, including light green mosaic patterns, and yellowish and dark red mosaic patterns. The other group of 10 plants included plants without symptoms (Table 1). Leaves from each plant were collected in May each year to evaluate the virus presence. Fruits were picked each year at the optimal maturity stage (end June–early July).

During 2019 and 2020, average monthly and annual air temperature (°C) and precipitation (mm) data were obtained from automatic weather sensors located near the orchards (Table 2). These data were sourced from the official Republic Hydrometeorological Service of Serbia.

### 2.2. RT-PCR Analysis

The presence of BlMaV in selected plants was evaluated using reverse transcription polymerase chain reaction (RT-PCR). Total nucleic acids (TNAs) from fresh leaf samples were extracted with a modified CTAB method [10]. Reverse transcription was performed with random hexamer primers and Maxima Reverse Transcriptase (Thermo Scientific, Waltham, MA, USA). PCR was performed with specific BlMaV-specific primers amplifying a 756 bp fragment from RNA3 [6]. Amplified PCR products were analyzed by 1.5% agarose gel electrophoresis, ethidium bromide staining, and visualized in Gel Doc EZ System with UV tray (Biorad laboratories, Hercules, CA, USA). All plants were further tested for the presence of other viruses infecting blueberries. For the presence of blueberry shock virus (*Ilarvirus* BSV, BlShV), blueberry leaf mottle virus (*Nepovirus myrtilli*, BLMoV), blueberry scorch virus (*Carlavirus vaccinii*, BlScV), blueberry shoestring virus (*Sobemovirus* BSSV, BSSV), tomato ringspot virus (*Nepovirus lycopersici*, ToRSV), tobacco ringspot virus (*Nepovirus nicotianae*, TRSV), and peach rosette mosaic virus (*Nepovirus persicae*, PRMV) plants were tested by ELISA test, following manufacturers’ protocols (Agdia Inc., Elkhart, IN, USA and BIOREBA AG, Reinach, Switzerland). To analyze the plants for the presence of blueberry red ringspot virus (*Soymovirus maculavaccinii*, BRRV), we performed PCR test as described earlier [11].

### 2.3. Preparation of Blueberry Extracts

Ground blueberry samples (10 g) were extracted with 25 mL of methanol using ultrasonic treatment. After 30 min of extraction, the mixture was centrifuged twice sequentially for 15 min at 3500 rpm. The resulting supernatant was filtered through a 0.22 μm Minisart filter prior to the determination of flavonoid, phenolic contents, and antioxidant capacity. The obtained extract was also utilized for UHPLC Q-ToF-MS analysis. The same extraction procedure was conducted using 25 mL of methanol/HCl (85:15, *v*/*v*) to obtain an extract for determining the anthocyanin content. All analyses were performed in triplicate, and the results are expressed as the mean ± standard deviation of three measurements.

### 2.4. Determination of Total Anthocyanins, Flavonoids, Phenolics, and Antioxidant Capacity

Monomeric anthocyanin content in the methanolic extracts was quantified using the pH differential method, as previously described [12,13]. A UV/VIS spectrophotometer (Agilent Technologies Cary Series 300, CA, USA) and a 1 cm path length disposable cell were used for spectral measurements at 510 and 700 nm. Results are expressed as mg of cyanidin-3-glucoside equivalents per 100 g of fresh weight (C3GE/100 g fw), using an extinction coefficient of 26,900 L/cm/mol and molecular weight of 449.2 g/mol.

Total flavonoid content was quantified using a colorimetric method [14], with results expressed as mg of catechin equivalents per 100 g fresh weight (mg CE/100 g fw). Fruit extract (1 mL) was mixed with 4 mL of distilled water. Subsequently, 0.3 mL of 5% NaNO_2_ solution was added, and the reaction was allowed to proceed for 5 min. Then, 0.3 mL of AlCl_3_ solution was added, followed by the addition of 2 mL of 1 M NaOH. The mixture was diluted to 10 mL with distilled water and thoroughly mixed. The absorbance was measured at 510 nm, using distilled water as the reference.

Total phenolic content was determined by a modified Folin–Ciocalteu colorimetric method, and values were reported as mg of gallic acid equivalents per 100 g of fresh weight (mg GAE/100 g fw) [14,15]. A 40 μL of fruit extracts or gallic acid standard solution (Merck, Darmstadt, Germany) was mixed with 3.16 mL of distilled water, whereupon 200 μL of Folin–Ciocalteu reagent (Merck) was added and allowed to stand for 8 min before 600 μL of 20% Na_2_CO_3_ solution was added. The solution was well-mixed and absorbance at 765 nm against an appropriate blank was determined after 2 h.

Antioxidant activity was determined using the DPPH method [16] and ABTS method [17], and the results were expressed as μmol trolox equivalents per 100 g fresh weight (μmol TE/100 g fw) and mmol trolox equivalents per 100 g fresh weight (mmol TE/100 g fw), respectively. Fruit extract (0.1 mL) was added to 3.9 mL of the DPPH solution in methanol (60 μM), and vortexed vigorously. After 30 min in the dark, absorbance was measured at 515 nm, using distilled water as the reference. As for the ABTS method, ABTS radical cation was generated by mixing 7 mM ABTS with 2.45 mM potassium persulfate and incubating for 12–16 h in the dark at room temperature. For the assay, 1 mL of ABTS radical solution was mixed with 1 mL of blueberry extract, and absorbance was measured at 734 nm after 6 min, using distilled water as the reference. The absorbances of the DPPH and ABTS radical solutions without fruit samples were also measured (blank), which is required for the calculation of radical scavenging activity.

### 2.5. UHPLC Q-ToF MS Analysis

High-performance liquid chromatography (Agilent 1290 Infinity Liquid Chromatography) coupled with a quadrupole and time-of-flight mass spectrometer (6530C Q-ToF-MS, Agilent Technologies, Inc., Santa Clara, CA, USA) was used for the analysis. Chromatographic separation was performed at 40 °C on a Zorbax C18 column (2.1 × 50 mm, 1.8 µm, Agilent Technologies, Inc., CA, USA). The mobile phase consisted of (A) ultrapure water and (B) 98% acetonitrile (MS grade), both containing 0.1% formic acid (MS grade). The flow rate was set to a constant 0.3 mL/min, and the injection volume was 5 µL. The gradient elution program was as follows: 0–2 min (98% A), 2–17 min (linear decrease to 2% A), followed by re-equilibration to the initial conditions (98% A) over the next 5 min to prepare the column for the subsequent injection.

The Q-ToF-MS system was equipped with an Agilent Jet Stream electrospray ionization (ESI) source operating in both negative and positive ionization modes. Blueberry extracts were analyzed in both ionization modes. The ion source operating parameters were as follows: nebulizer pressure 45 psi, drying gas temperature 225 °C with a flow rate of 8 L/min, sheath gas temperature 300 °C with a flow rate of 10 L/min, capillary voltage 2500 V, fragmentor voltage 175 V, skimmer voltage 65 V, and RF peak voltage on the octopole 750 V. The Q-ToF-MS system acquired spectra in the m/z range of 100–1700, at a scan rate of 2 Hz. Data-dependent acquisition (DDA) was employed for suspect screening using the AutoMS/MS acquisition mode. The parameters for the Auto MS/MS mode were *m*/*z* range = 100–1700, scan rate of 1 spectrum/sec, and a fixed collision energy of 30 eV.

Agilent MassHunter software was used for data acquisition, instrument control, and spectral evaluation. Bioactive compounds were identified based on their accurate *m*/*z* (monoisotopic mass), MS fragmentation patterns, and available literature data [18,19,20,21]. Some of the identified phenolic compounds were further confirmed by direct comparison with available standards (chlorogenic acid, kaempferol, quercetin, isorhamnetin, myricetin, rutin, naringenin and procyanidin B2; standards purchased from Chem Faces, >98% purity, Wuhan, Hubei, China). To further clarify the identification of unknown compounds, mass spectra for all identified compounds are grouped and presented in Figures S1–S4. Fragmentation patterns (MS/MS spectra) were exported from AgilentMassHunter software (AgilentMassHunter Qualitative Analysis 10.0). Exact masses of the compounds were calculated using ChemDraw software (version 12.0, CambridgeSoft, Cambridge, MA, USA). CAS SciFinder (https://scifinder.cas.org) (accessed on 14 July 2025.) was used for the structural- and formula-based search of bioactive compounds.

In this study, untargeted UHPLC Q-ToF MS analysis was primarily used for profiling and identification of all unknown bioactive compounds in blueberry samples, which were further correlated with the secondary metabolism of healthy and infected blueberry plants. Due to the reasons mentioned, the robustness of untargeted analysis data and limitations for quantitative analysis, statistical analysis included and used normalized peak areas (relative change-values, %), to indicate differences in the same compound between healthy and infected blueberry samples from two years (Table S1). Peak areas for each individually identified compound can be considered and compared between samples, as all samples were processed under identical conditions (same extraction protocol, identical injection volumes, and chromatographic settings). Peak areas for each identified compound in blueberry samples were exported from AgilentMassHunter software.

### 2.6. Statistical Analysis

A two-factorial experimental design using ANOVA and Tukey’s multiple comparison tests was used to analyze the data, using Statistica 7 (StatSoft, Inc., Tulsa, OK, USA). The viral status of the plant (BlMaV-infected, BlMaV-free), and harvest year (2019, 2020) were taken as the factors of variation. Principal component analysis (PCA), based on the content of 11 individual phenolic compounds and 2 groups of compounds, was performed, and PCA was designed. The correlations between observed compounds were graphically presented using color correlation analysis performed in *R* software version 4.0.3 (64-bit).

## 3. Results

### 3.1. BlMaV Detection

**Figure 1:**
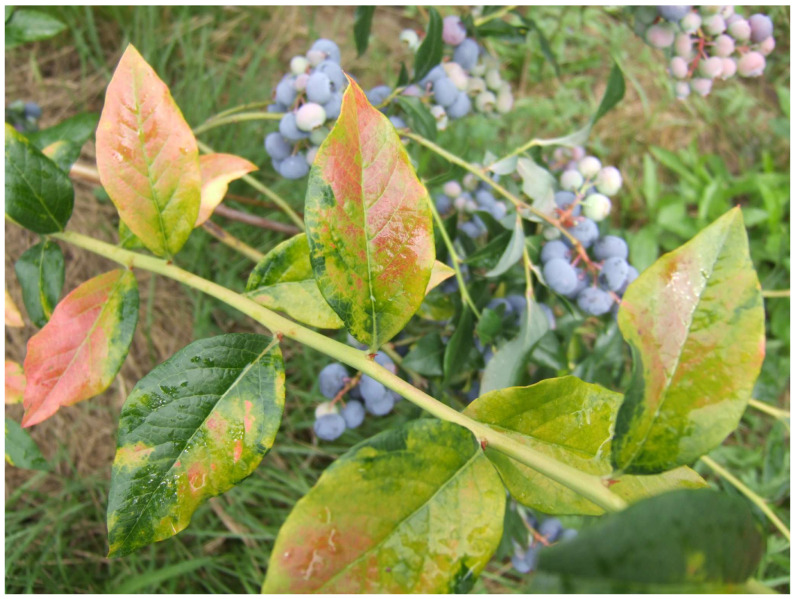
Symptoms of the blueberry mosaic disease on ‘Duke’ leaves.

A total of 20 blueberry ‘Duke’ plants were analyzed for BlMaV presence by RT-PCR. The analysis confirmed the presence of BlMaV in all samples with mosaic symptoms (10 samples) (Figure 1) and confirmed BlMaV-free status in asymptomatic samples (10 samples). The ELISA and PCR analysis confirmed the absence of all other tested viruses (BlShV, BLMoV, BlScV, BSSV, ToRSV, TRSV, PRMV, and BRRV), excluding their influence on the investigated properties.

### 3.2. Quantification of Total Anthocyanins, Flavonoids, and Phenolics, and Determination of Antioxidative Capacity

The total amounts of certain phenolic groups, and antioxidative capacity in healthy and viral blueberry samples over two successive years are presented in Table 3. These results are comparable with previously reported data for ‘Duke’ blueberry cultivar [22,23,24]. Viral infection had no influence on total phenolic content and, consequently, on the antioxidative capacity. Nevertheless, BlMaV infection exerted a detectable influence on the flavonoid content, restricted to 2019. The influence of the harvest year was significantly more pronounced, most likely due to the different environmental conditions (temperature and rainfall) during 2019 and 2020. For instance, the total amounts of anthocyanins in B19− and B20− were 112.06 and 129.57, respectively, which is an evident influence of harvest year on healthy blueberry samples. However, the total anthocyanins content between infected and healthy blueberries from the same harvest year was not significantly different. Finally, total anthocyanins and flavonoid content reflects the cumulative outcome of all individual anthocyanins and flavonoids, while individual compounds may not always follow this overall trend. It should also be noted that the UHPLC-QToF-MS approach provides considerably higher specificity and accuracy for individual anthocyanins and other flavonoids than the colorimetric methods typically applied for total anthocyanins and flavonoids. Therefore, the higher total anthocyanin levels or lower total flavonoid content observed in infected fruit should not be considered contradictory to the individual data, but rather as a difference in the distribution of individual anthocyanins and flavonoids in general.

Taking into account only these results, one can conclude that BlMaV infection had very restricted influence on the phenolic profile of ‘Duke’ blueberry samples, which may even suggest that the ‘Duke’ cultivar is resistant to BlMaV infection. However, in order to validate or refute such a claim, we found it essential to conduct a detailed polyphenolic analysis of the fruit samples.

### 3.3. Identification of Phenolic Compounds

UHPLC Q-ToF MS analysis has primarily been used for the detection, identification, and characterization of bioactive compounds (especially phenolic compounds) extracted from blueberry fruit, to provide insight into the metabolite profile accumulated in blueberry fruits and to clarify their connection with the secondary metabolism of healthy and infected blueberry plants. The MS base peak chromatograms of the analyzed extracts, in both negative and positive ionization modes, are depicted in the Supplementary Materials (Figure S5). The peaks of all identified compounds were extracted from the MS base peak chromatograph (Figure S5; for peak annotation, see retention times in Table 4), considering the monoisotopic mass for each precursor ion. To achieve the most reliable and the most precise UHPLC Q-ToF MS identification of unknown bioactive compounds, all aspects of mass spectrometric analysis were taken into account [25]. In this case, untargeted analysis revealed 42 phenolic compounds in blueberry samples, including 12 phenolic acids and their derivatives, 7 flavonol aglycones, 10 flavonol glycosides and acyl derivatives, one flavanone (naringenin), one other phenolic compound (vanilloloside), one proanthocyanidin (procyanidin B2), and 10 anthocyanins (Table 4). In addition, three terpenoids and abscisic acid (a plant hormone) were found and characterized as nonphenolic compounds. Among the detected phenolic compounds, only several were confirmed by direct comparison with available standards (Category 1#––confirmed compounds; these compounds are marked in Table 4). Other compounds were identified based on their *m*/*z* exact mass (monoisotopic mass) which was used to predict formulas of unknown compounds and typical MS fragments (Category #2––tentatively identified compounds), and further clarified by comparison with available literature data.

Most of the identified compounds were previously reported in blueberry fruits of various cultivars [18,19,21,26,27,28,29,30,31]. Moreover, representative references concerning the previous identification of the mentioned compounds in various blueberry fruits or leaves are listed in Table 4. 

All identified phenolic acids exist as methyl derivatives or as esters with quinic acid. Among the phenolic acids, gallic acid was found only in the form of methyl derivatives. Methyl gallate (**1**), dimethyl-digallate (**3**), and tetramethyl-digallate (**8**) were detected based on their characteristic monoisotopic masses and fragments obtained by the loss of methyl group(s) or galloyl moiety. Other identified compounds included caffeic acid hexoside (**2**), caffeoylquinic acid isomers (**4–6**), dicaffeoylquinic acid (**11**), caffeoylquinic acid dimer (**12**), and caffeoylquinic acid derivatives (**7** and **10**). Caffeic acid hexoside was identified based on typical fragments at 179 *m*/*z* (caffeic acid moiety), 161 *m*/*z* (loss of H_2_O), and 135 *m*/*z* (loss of CO_2_). This compound was selectively detected in blueberry samples (B19− and B20−). Caffeoylquinic acid and its derivatives showed the same fragments corresponding to deprotonated caffeic acid (179, 161, 135 *m*/*z*) and/or quinic acid (191, 173, 155, 127, or 111 *m*/*z*). Caffeoylquinic acid was found in the form of three isomers, which have the same mass of precursor ions and similar MS/MS fragmentation patterns with identical fragments, but different elution times. One of them (**5**) was recognized as chlorogenic acid (3-caffeoylquinic acid) by comparison with an available standard. Dicaffeoylquinic acid was recognized based on the unique mass of precursor ion (*m*/*z* 515.1183; C_25_H_23_O_12_^–^) and more specific fragments at 353 *m*/*z* and 335 *m*/*z* obtained by the loss of a caffeoyl moiety (−162 Da) and H_2_O (−18 Da). Caffeoylquinic acid dimer showed typical fragmentation [32], with main fragments at 513 and 321 *m*/*z* obtained by sequential loss of quinic acids. This compound was found in all samples except in B19+. Compound **10** was identified as caffeoyl coumaroylquinic acid, with typical fragments corresponding to the coumaroyl moiety (163 and 119 *m*/*z*), caffeoyl moiety, and deprotonated quinic acid. In addition to the compounds mentioned, diferulic acid was identified and detected only in B20+. This compound showed typical fragments obtained by cleavage of ferulic acid (178, 161, 133, and 134 *m*/*z*). Ancillotti et al. [33] reported a very similar phenolic acid profile across different *Vaccinium* berry species, highlighting the predominance of caffeic acid derivatives.

Among flavonoids, flavonols and anthocyanins were predominantly identified, in the forms of aglycones, glycosides, and/or acyl derivatives. Flavonol aglycones (**13–19**) exhibited typical fragments produced by the retro Diels–Alder (RDA) reaction (C-ring cleavage) (^0,4^A^–^; ^1,2^A^–^; ^1,3^A^–^; or ^1,2^B^–^) and/or fragments obtained by neutral losses of CO, CO_2_, H_2_O, CH_2_O, H_2_O, CH_3,_ etc. Kaempferol, quercetin, isorhamnetin, and myricetin were also confirmed using available standards. Compounds **17** and **18** are isomers with the identical monoisotopic masses and molecular formulas but different structures (differing positions of methyl and hydroxyl groups), MS/MS fragmentation patterns and elution times. Compound **18** was identified as laricitrin, with characteristic fragments at 316 *m*/*z* (loss CH_3_), 151 *m*/*z* (^1,3^A^–^;) and 179 *m*/*z* (^1,2^A^–^). On the contrary, compound **17** was recognized as patuletin, with main fragments at 299, 271, 255, 243, 227 and 215 *m*/*z*, resulting from neutral loss of H_2_O, CH_3_, CO and/or [O]. Syringetin exhibited a similar MS/MS fragmentation pattern as laricitrin (Figure S2), with main fragments at 330 *m*/*z* and 315 *m*/*z*, produced by sequential loss of two methyl groups. Flavonol glycosides (**20–24**, and **26**) were identified based on monoisotopic mass and typical fragments corresponding to deprotonated flavonol aglycones, formed by loss of a pentosyl (−132 Da) or hexosyl (−162 Da) unit. Compounds **25** and **27** were identified as quercetin 3-*O*-(6″-acetyl)hexoside and isorhamnetin 3-*O*-(6″-acetyl)hexoside, respectively, with main fragments corresponding to deprotonated quercetin (300 *m*/*z*; [Y_0_—H]^−^) and isorhamnetin (314 *m*/*z*; [Y_0_—H]^−^). Compounds **28** and **29** were detected as quercetin 3-*O*-(6″-*O*-rhamnosyl)hexoside (such as rutin) and isorhamnetin 3-*O*-(6″-*O*-rhamnosyl)hexoside (such as narcissin), with main fragments at 301/300 *m*/*z* and 315/314 *m*/*z* ([Y0]^–^/[Y_0_—H]^−^), obtained by loss of rhamnosyl-hexoside (−308 Da; 1→6 interglycosidic linkage between sugars). Except for kaempferol and myricetin 3-*O*-pentoside, all other flavonols were found in all analyzed blueberry samples. In addition, aforementioned flavonol glycosides and acyl derivatives (except **27**) have previously been reported in various blueberry cultivars [18,31]. In addition to the phenolic compounds mentioned, naringenin (**30**) and procyanidin B-type dimer (such as procyanidin B2) (**32**) were identified and confirmed by comparison with standards.

Anthocyanins were identified in a positive ionization mode as glycosides and acyl derivatives of peonidin (*m*/*z* 301), delphinidin (*m*/*z* 303), petunidin (*m*/*z* 317), and/or malvidin (*m*/*z* 331). In all analyzed blueberry extracts (samples), hexosides (galactose or glucose) and pentosides of delphinidin, petunidin, and malvidin were identified (**33–38**). In addition, acetyl-hexosides (6″-acetyl-hexoside) of peonidin, delphinidin, petunidin, and malvidin (**39–42**) were also confirmed. However, peonidin 3-*O*-(6″-acetyl)hexoside (**39**) and delphinidin 3-*O*-(6″-acetyl)hexoside (**40**) were selectively detected in the analyzed blueberry samples (Table S1). Differences in the distribution of individual anthocyanins in blueberry fruits can be attributed to the exposure of the plant to stressful conditions. The mentioned anthocyanins and acetyl-derivatives are typical compounds derived from blueberries, as previously reported by other authors [21,27,34]. For example, Kim et al. [34] identified 22 anthocyanin derivatives, consisting of mono-glycosides and acetyl-glycosides, attached to previously mentioned aglycones, in nine cultivars of highbush blueberries. Further, Wang et al. [27] analyzed the anthocyanin compositions of 62 blueberry cultivars using the UPLC–MS technique, and identified 30 anthocyanins derived from five anthocyanidins, including five xylosides. The most comprehensive analysis of the anthocyanin profile was conducted by Ancillotti et al. [33], who identified as many as 64 derivatives.

The presence of pentacyclic terpenoid (compounds 43–45) in berry fruits is well-documented [35,36]. In these studies, three pentacyclic terpenoids (**43–45**) were selectively detected in blueberry samples, without clear characterization. These compounds were identified based on typical fragments resulting from the loss of H_2_O (−18 Da) and CO_2_ (−44 Da). Abscisic acid (**46**) was found in all analyzed samples. This compound is a plant growth regulator whose increased content is associated with the onset of ripening, and likely plays a key role in the ripening process and may contribute to the regulation of flavonoid biosynthesis in blueberries [37].

**Table 4:** Identification and characterization of phenolic and nonphenolic (pentacyclic terpenoids and abscisic acid) compounds in BlMaV-infected and virus-free blueberry samples, using UHPLC Q-ToF MS (untargeted analysis).

No.	Compounds Name	RT (min)	Formula	Calculated Mass	*m*/*z* Exact Mass	mDa	Fragments (MS^2^)	Ref **
Phenolic acids and derivatives								
1	Methyl gallate	5.64	C_8_H_7_O_5_^−^	183.0293	183.0295	0.15	**124.0159(100)**, 125.019, 106.0054	[38]
2	Caffeic acid hexoside	5.50	C_15_H_17_O_9_^−^	341.0873	341.0865	−0.76	**135.0444(100)**, 179.0338, 161.0228	[39]
3	Dimethyl-digallate	7.39	C_16_H_13_O_9_^−^	349.056	349.056	0.04	**165.0183(100)**, 137.0236, 123.0081, 151.0028, 183.0297, 197.0445	-
4	Caffeoylquinic acid is. I	4.81	C_16_H_17_O_9_^−^	353.0873	353.0866	−0.66	**191.0549(100)**, 135.0443, 179.0339, 161.0231, 173.0450, 127.0398, 111.0441	[40]
5	Caffeoylquinic acid is. II (Chlorogenic acid) *	6.34	C_16_H_17_O_9_^−^	353.0873	353.0866	−0.66	**191.0550(100)**, 173.0446, 161.0235, 135.0444, 127.0395	Std.
6	Caffeoylquinic acid is. III	7.05	C_16_H_17_O_9_^−^	353.0873	353.0866	−0.66	**191.0550(100)**, 173.0435, 161.0235, 127.0395, 111.0442	[40]
7	Caffeoylquinic acid methyl ester	7.65	C_17_H_19_O_9_^−^	367.1029	367.1026	−0.31	**135.0446(100)**, 179.0342, 161.0234, 191.0551	[41]
8	Tetramethyl-digallate	9.07	C_18_H_17_O_9_^−^	377.0873	377.087	−0.26	**165.0184(100)**, 137.0231, 121.0186, 151.0034, 190.9980, 315.0131, 330.0345, 166.0219	-
9	Diferulic acid	11.56	C_20_H_17_O_8_^−^	385.0923	385.092	−0.34	**193.0491(100)**, 134.0366, 133.0302, 161.0230, 178.0253, 317.0350	-
10	Caffeoyl coumaroylquinic acid	8.77	C_25_H_23_O_11_^−^	499.124	499.1231	–0.94	**163.0392(100)**, 191.0550, 173.0442, 155.0341, 135.0446, 119.0495, 337.0907, 179.0337	[42]
11	Dicaffeoylquinic acid	8.18	C_25_H_23_O_12_^−^	515.119	515.1183	−0.65	**179.0339(100)**, 191.0550, 173.0445, 161.0233, 135.0444, 335.0766, 353.0860	[42]
12	Caffeoylquinic acid dimer	7.19	C_32_H_33_O_18_^−^	705.1667	705.1652	−1.49	**513.1014(100)**, 339.0483, 191.0545, 321.0375, 495.0926	-
Flavonol aglycones								
13	Kaempferol *	10.22	C_15_H_9_O_6_^−^	285.0399	285.0397	−0.21	**285.0390(100)**, 257.0425, 229.0488, 211.0404, 185.0587, 143.0528, 151.0064	Std.; [43]
14	Quercetin *	9.50	C_15_H_9_O_7_^−^	301.0348	301.0345	−0.33	**151.0029(100)**, 121.0290, 107.0135, 164.0109, 178.9975, 229.0487, 245.0438, 271.0234	Std.; [43]
15	Isorhamnetin *	10.42	C_16_H_11_O_7_^−^	315.0505	315.0497	−0.78	**300.0261(100)**, 151.0035, 107.0141, 137.0233, 164.0108, 178.9993, 203.0324, 227.0339, 259.0225	Std.; [44]
16	Myricetin *	8.69	C_15_H_9_O_8_^−^	317.0297	317.0296	−0.14	**151.0029(100)**, 137.0237, 107.0135, 125.0239, 165.0182, 178.9977, 227.0338, 243.0280, 271.0233	Std.; [43]
17	Patuletin	9.06	C_16_H_11_O_8_^−^	331.0454	331.0453	−0.09	**243.0285(100)**, 299.0176, 271.0230, 255.0273, 227.0341, 215.0335, 199.0389, 183.0447, 171.0443, 143.0498	-
18	Laricitrin	9.58	C_16_H_11_O_8_^−^	331.0454	331.0453	−0.09	**151.0044(100)**, 316.0206, 299.0171, 271.0230, 259.0236, 178.9978, 164.0104, 287.0184, 136.0160, 107.0132	[39]
19	Syringetin	10.38	C_17_H_13_O_8_^−^	345.061	345.0602	−0.84	**315.0134(100)**, 287.0184, 330.0364, 345.0603, 301.0340, 259.0235, 271.0237, 203.0336, 151.0029	[45]
Flavonol glycosides and acyl derivatives								
20	Quercetin 3-*O*-pentoside	8.16	C_20_H_17_O_11_^−^	433.0771	433.0767	−0.39	**300.0259(100)**, 271.0234, 255.0289, 178.9989, 151.0032	[18]
21	Myricetin 3-*O*-pentoside	5.97	C_20_H_17_O_12_^−^	449.072	449.0714	−0.60	**449.0702(100)**, 299.0172, 317.0280, 271.0215, 190.9972	[18]
22	Quercetin 3-*O*-hexoside	7.89	C_21_H_19_O_12_^−^	463.0877	463.0871	−0.55	**300.0260(100)**, 301.0323, 463.0864, 271.0234, 255.0284, 151.0030, 178.9987	[18]
23	Myricetin 3-*O*-hexoside	7.44	C_21_H_19_O_13_^−^	479.0826	479.084	1.43	**316.0207(100)**, 317.0261, 271.0234, 187.0184, 479.0810, 178.9980	[18]
24	Laricitrin 3-*O*-hexoside	8.06	C_22_H_21_O_13_^−^	493.0982	493.0977	−0.52	**330.0365(100)**, 331.0419, 315.0133, 300.0260, 287.0514, 178.9973, 151.0039, 433.0758	[18]
25	Quercetin 3-*O*-(6″-acetyl)hexoside	8.49	C_23_H_21_O_13_^−^	505.0982	505.0978	−0.42	**300.0262(100)**, 344.0518, 271.0234, 178.9974, 151.0025, 463.0861	[31]
26	Syringetin 3-*O*-hexoside	8.39	C_23_H_23_O_13_^−^	507.1139	507.1125	−1.37	**344.0521(100)**, 507.1112, 345.0574, 387.0699, 329.0300, 316.0569, 301.0403, 273.0381, 151.0031	[18]
27	Isorhamnetin 3-*O*-(6″-acetyl)hexoside	8.99	C_24_H_23_O_13_^−^	519.1139	519.113	−0.87	**314.0418(100)**, 519.1125, 315.0462, 299.0203, 285.0393, 271.0241, 257.0443, 243.0289, 151.0025, 357.0595	-
28	Quercetin 3-*O*-(6″-*O*-rhamnosyl)hexoside (such as Rutin) *	7.74	C_27_H_29_O_16_^−^	609.1456	609.145	−0.56	**300.0261(100)**, 609.1435, 301.0329, 271.0235, 151.003, 178.9975, 343.0431	Std.; [18]
29	Isorhamnetin 3-*O*-(6″-*O*-rhamnosyl)hexoside (such as Narcissin)	8.22	C_28_H_31_O_16_^−^	623.1612	623.1608	−0.41	**315.049(100)**, 623.1592, 314.0416, 300.0249, 271.0241, 151.0022, 357.0595	[31]
Other phenolic compounds								
30	Naringenin *	10.04	C_15_H_11_O_5_^−^	271.0606	271.0603	−0.35	**119.0499(100)**, 107.0132, 151.0024, 161.0590, 187.0388, 229.0458, 245.0477	Std.; [46]
31	Vanilloloside	3.95	C_14_H_19_O_8_^−^	315.108	315.108	0.01	**123.0445(100)**, 153.0547, 124.0478	**-**
32	Procyanidin B-type dimer (such as Procyanidin B2) *	6.04	C_30_H_25_O_12_^−^	577.1346	577.1335	−1.10	**289.0700(100)**, 407.0752, 125.0237, 137.0243, 161.0241, 245.0796, 273.0388, 339.0842, 381.0951, 425.0876, 451.1001	Std.; [38]
Anthocyanins								
33	Delphinidin 3-*O*-pentoside	6.47	C_20_H_19_O_11_^+^	435.0927	435.0919	−0.84	**303.0488(100)**, 304.0523, 305.0543	[21]
34	Petunidin 3-*O*-pentoside	6.74	C_21_H_21_O_11_^+^	449.1084	449.1075	−0.89	**317.0644(100)**, 318.068, 287.0535, 302.0409	[21]
35	Malvidin 3-*O*-pentoside	7.07	C_22_H_23_O_11_^+^	463.124	463.1232	−0.84	**331.0802(100)**, 332.0835, 301.0695, 315.0488, 287.0534	[21]
36	Delhinidin 3-*O*-hexoside	6.19	C_21_H_21_O_12_^+^	465.1033	465.1025	−0.80	**303.0488(100)**, 304.0522, 305.0543	[21]
37	Petunidin 3-*O*-hexoside	6.6	C_22_H_23_O_12_^+^	479.119	479.1182	−0.75	**317.0645(100)**, 318.0677, 302.0409	[21]
38	Malvidin 3-*O*-hexoside	6.93	C_23_H_25_O_12_^+^	493.1346	493.1338	−0.8	**331.0802(100)**, 332.0835, 315.0486, 287.0536	[21]
39	Peonidin 3-*O*-(6″-acetyl)hexoside	7.8	C_24_H_25_O_12_^+^	505.1346	505.1343	−0.30	**301.0692(100)**, 302.0731, 303.0702, 286.0457	[21]
40	Delphinidin 3-*O*-(6″-acetyl)hexoside	7.32	C_23_H_23_O_13_^+^	507.1139	507.1131	−0.77	**303.0487(100)**, 304.0521, 305.0547	[21]
41	Petunidin 3-*O*-(6″-acetyl)hexoside	7.48	C_24_H_25_O_13_^+^	521.1295	521.1286	−0.92	**317.0643(100)**, 318.068, 302.0406	[21]
42	Malvidin 3-*O*-(6″-acetyl)hexoside	7.68	C_25_H_27_O_13_^+^	535.1452	535.1444	−0.77	**331.0801(100)**, 332.0835, 315.0486	[21]
	Other compounds (Terpenoids)							
43	Pentacyclic terpenoid (like Maslinic or Pomolic acid)	14.79	C_30_H_47_O_4_^−^	471.3474	471.3472	−0.23	**471.3460(100)**, 453.3342, 427.3566, 409.3457	[19]
44	Pentacyclic terpenoid I (like Arjunolic, Euscaphic or Rotundic acid)	12.58	C_30_H_47_O_5_^−^	487.3423	487.3415	−0.85	**487.3396(100)**, 469.3305, 437.3053, 425.3392, 405.3107, 393.3127, 443.3483	[19]
45	Pentacyclic terpenoid II (like Arjunolic, Euscaphic or Rotundic acid)	12.92	C_30_H_47_O_5_^−^	487.3423	487.3415	−0.85	**487.3405(100)**, 469.3288, 425.3394, 407.3258, 443.3536, 393.3111	[19]
	Other compounds (Plant hormone)							
46	Abscisic acid	9.31	C_15_H_19_O_4_^−^	263.1283	263.1283	−0.03	**203.1068(100)**, 219.1372, 289.0910, 153.0911, 136.0521, 122.0367, 125.0605, 148.0525	[47]

* Compounds confirmed by direct comparison with available standards. ** Representative references related to the previous identification of mentioned compounds in various blueberry fruits or leaves. Std.—Standard.

### 3.4. Color Correlation Analysis

**Figure 2:**
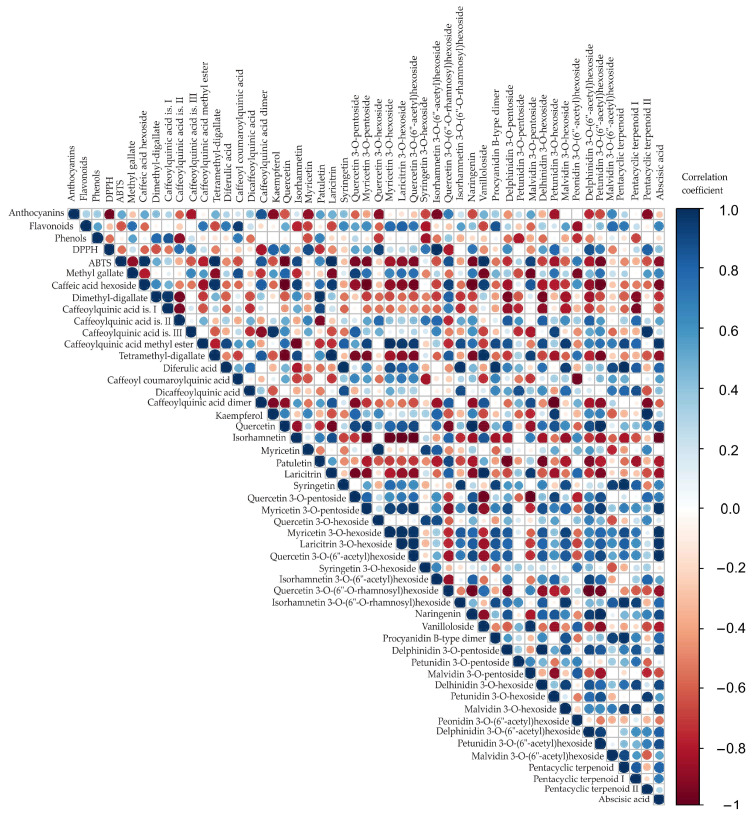
Color correlation diagram between total anthocyanin, flavonoid, and phenolic contents, and antioxidant capacity (DPPH and ABTS) and observed compounds for blueberry samples.

Correlation analysis was conducted to explore potential relationships among the 46 detected compounds in the blueberry samples (Table 4). Figure 2 provides a visual representation of these correlations, with circle color indicating the direction (red: negative; blue: positive) and circle size reflecting the magnitude of the correlation (larger circles correspond to stronger correlations) [48]. This visualization facilitates the identification of compound clusters and underlying patterns in the dataset. By highlighting strong positive or negative correlations, this approach can help to reveal which groups of bioactive compounds may be biosynthetically or functionally related, providing insights into the phytochemical composition of the samples.

The correlation analysis (Figure 2) revealed numerous statistically significant associations (*p* < 0.05) among total anthocyanin, flavonoid, and phenolic contents, and antioxidant capacity (DPPH) and the 46 detected metabolites, particularly within biosynthetically related groups such as phenolic acids, flavonol aglycones, glycosides, anthocyanins, and terpenoids. The correlation analysis revealed several statistically significant associations among the studied variables of bluberry samples (*r* > 0.99, *p* < 0.05), suggesting that these bioactive groups contribute accordingly to the overall antioxidant potential of bluberry samples. Anthocyanins showed a strong negative correlation with DPPH (*r* = −0.968, *p* = 0.033), indicating that higher anthocyanin levels were related to stronger radical scavenging activity. Flavonoids were positively associated with dicaffeoylquinic acid (*r* = 0.955, *p* = 0.045), suggesting a structural relationship between total flavonoid content and this specific phenolic derivative. ABTS displayed highly significant positive correlations with caffeic acid hexoside (*r* = 0.985, *p* = 0.015) and tetramethyl-digallate (*r* = 0.999, *p* = 0.002), while also correlating strongly with syringetin (*r* = 0.993, *p* = 0.007) and procyanidin B-type dimer (*r* = 0.982, *p* = 0.018). These findings highlight ABTS as a more sensitive antioxidant assay to phenolic composition compared with DPPH, which correlated negatively with most polyphenols. Phenols exhibited positive but statistically non-significant associations with most caffeoylquinic acid derivatives, indicating possible structural but not functional linkages under the tested conditions. Furthermore, a strong negative correlation was observed between methyl gallate and vanilloloside (*r* = −0.955, *p* < 0.01) as well as malvidin 3-*O*-pentoside (*r* = −0.963, *p* < 0.01). Caffeic acid hexoside showed strong positive correlation with tetramethyl-digallate (*r* = 0.977, *p* < 0.01) and laricitrin (*r* = 0.959, *p* < 0.01), while on the other hand, it showed negative correlation with quercetin (*r* = −0.990, *p* < 0.001), myricetin 3-*O*-pentoside (*r* = −0.988, *p* < 0.01), quercetin 3-*O*-(6″-acetyl)hexoside (*r* = −0.963, *p* < 0.01), naringenin (*r* = −0.981, *p* < 0.01), and petunidin 3-*O*-(6″-acetyl)hexoside (*r* = −0.983, *p* < 0.01). Dimethyl-digallate showed a strong positive association with caffeoylquinic acid is. I (*r* = 0.978, *p* < 0.01) and patuletin (*r* = 0.980, *p* < 0.01), while showing a notable inverse correlation with caffeoylquinic acid is. II (*r* = −0.953, *p* < 0.01), delphinidin 3-*O*-pentoside (*r* = −0.951, *p* < 0.01), and delhinidin 3-*O*-hexoside (*r* = −0.953, *p* < 0.01). Caffeoylquinic acid is. III is positively correlated with kaempferol (*r* = 0.979, *p* < 0.01) and pentacyclic terpenoid II (*r* = 0.979, *p* < 0.01). Caffeoylquinic acid methyl ester strongly negatively correlated with isorhamnetin (*r* = −0.970, *p* < 0.01), while positively correlating with myricetin 3-*O*-hexoside (*r* = 0.995, *p* < 0.01), laricitrin 3-*O*-hexoside (*r* = 0.966, *p* < 0.01), quercetin 3-*O*-(6″-acetyl)hexoside (*r* = 0.955, *p* < 0.01), malvidin 3-*O*-hexoside (*r* = 0.956, *p* < 0.01), and abscisic acid (r = 0.974, *p* < 0.01). Tetramethyl-digallate is negatively correlated with quercetin (*r* = −0.975, *p* < 0.01) and petunidin 3-*O*-(6″-acetyl)hexoside (*r* = −0.953, *p* < 0.01), while at the same time positively correlated with laricitrin (*r* = 0.997, *p* < 0.01) and vanilloloside (*r* = 0.989, *p* < 0.01). Diferulic acid is statistically significant positively corelated with syringetin (*r* = 0.981, *p* < 0.01), isorhamnetin 3-*O*-(6″-*O*-rhamnosyl)hexoside (*r* = 0.975, *p* < 0.01), procyanidin B-type dimer (*r* = 0.970, *p* < 0.01). Caffeoyl coumaroylquinic acid is negatively correlated with peonidin 3-*O*-(6″-acetyl)hexoside (*r* = −0.974, *p* < 0.01), while caffeoylquinic acid dimer is negatively correlated with petunidin 3-*O*-hexoside (*r* = −0.997, *p* < 0.01). Kaempferol is positively correlated with petunidin 3-*O*-hexoside (*r* = 0.951, *p* < 0.01). Quercetin is negatively correlated with laricitrin (*r* = −0.962, *p* < 0.01), while positively correlating with myricetin 3-*O*-pentoside (*r* = 0.959, *p* < 0.01), naringenin (*r* = 0.992, *p* < 0.01), and petunidin 3-*O*-(6″-acetyl)hexoside (*r* = 0.996, *p* < 0.01). Isorhamnetin is in highly negative correlation with myricetin 3-*O*-pentoside (*r* = −0.971, *p* < 0.01), myricetin 3-*O*-hexoside (*r* = −0.988, *p* < 0.01), laricitrin 3-*O*-hexoside (*r* = −0.998, *p* < 0.01), quercetin 3-*O*-(6″-acetyl)hexoside (*r* = −0.996, *p* < 0.01), and abscisic acid (*r* = −0.987, *p* < 0.01). Myricetin showed strong positive correlation with quercetin 3-*O*-hexoside (*r* = 0.986, *p* < 0.01); on the other hand, patuletin exhibited strong negative correlation with delphinidin 3-*O*-pentoside (*r* = −0.979, *p* < 0.01) and delhinidin 3-*O*-hexoside (*r* = −0.964, *p* < 0.01). Laricitrin was negatively correlated with quercetin 3-*O*-pentoside (*r* = −0.966, *p* < 0.01), while at the same time, it showed positive correlation with vanilloloside (*r* = 0.997, *p* < 0.01) and malvidin 3-*O*-pentoside (*r* = 0.954, *p* < 0.01). Syringetin showed statistically significant positive correlation with isorhamnetin 3-*O*-(6″-O-rhamnosyl)hexoside (*r* = 0.968, *p* < 0.01) and pentacyclic terpenoid (*r* = 0.981, *p* < 0.01), while the opposite correlation is noticed between quercetin 3-*O*-pentoside and vanilloloside (*r* = −0.977, *p* < 0.01), and malvidin 3-*O*-pentoside (*r* = −0.994, *p* < 0.01). On the other hand, myricetin 3-*O*-pentoside showed positive correlation with laricitrin 3-*O*-hexoside (*r* = 0.956, *p* < 0.01), quercetin 3-*O*-(6″-acetyl)hexoside (*r* = 0.988, *p* < 0.01), naringenin (*r* = 0.954, *p* < 0.01), petunidin 3-*O*-(6″-acetyl)hexoside (*r* = 0.953, *p* < 0.01), and abscisic acid (*r* = 0.985, *p* < 0.01). Furthermore, myricetin 3-*O*-hexoside showed strong positive correlation with laricitrin 3-*O*-hexoside (*r* = 0.987, *p* < 0.01), quercetin 3-*O*-(6″-acetyl)hexoside (*r* = 0.974, *p* < 0.01), and abscisic acid (*r* = 0.980, *p* < 0.01). There is a strong positive correlation between abscisic acid and laricitrin 3-*O*-hexoside and quercetin 3-*O*-(6″-acetyl)hexoside (*r* = 0.975, *p* < 0.01 and *r* = 0.991, *p* < 0.01, respectively). Laricitrin 3-*O*-hexoside is also positively correlated with quercetin 3-*O*-(6″-acetyl)hexoside (*r* = 0.989, *p* < 0.01). A significant negative correlation can be observed between quercetin 3-*O*-(6″-*O*-rhamnosyl)hexoside and naringenin (*r* = −0.976, *p* < 0.01), delphinidin 3-*O*-(6″-acetyl)hexoside (*r* = −0.992, *p* < 0.01), and petunidin 3-*O*-(6″-acetyl)hexoside (*r* = −0.966, *p* < 0.01). Isorhamnetin 3-*O*-(6″-*O*-rhamnosyl)hexoside showed positive correlation with malvidin 3-*O*-hexoside (*r* = 0.969, *p* < 0.01) and pentacyclic terpenoid (*r* = 0.975, *p* < 0.01). There is also a high positive correlation between the following compounds: naringenin and petunidin 3-*O*-(6″-acetyl)hexoside (*r* = 0.999, *p* < 0.01), vanilloloside and delphinidin 3-*O*-pentoside (*r* = 0.972, *p* < 0.01), procyanidin B-type dimer and pentacyclic terpenoid (*r* = 0.970, *p* < 0.01), delphinidin 3-*O*-pentoside and delhinidin 3-*O*-hexoside (*r* = 0.993, *p* < 0.01), malvidin 3-*O*-pentoside and vanilloloside (*r* = 0.972, *p* < 0.01), and petunidin 3-*O*-hexoside and pentacyclic terpenoid II (*r* = 0.951, *p* < 0.01).

### 3.5. Principal Component Analysis

In order to present the results in a more illustrative manner, principal component analysis (PCA) was applied to determine how the infected and healthy blueberry ‘Duke’ samples over two consecutive harvest years were grouped based on the relative concentrations of 46 detected individual compounds. In this work, the actual concentrations of detected compounds were not determined. Therefore, the peak areas were calculated, and for each compound, the highest peak among the four samples (B19−, B19+, B20−, B20+) was set as 100%. The peak areas of the remaining three samples were then expressed as percentages relative to the highest peak. The results of these calculations are presented in Table S1. Based on the normalized peak areas (expressed as percentages relative to the highest peak for each compound), a PCA was performed in order to evaluate the similarities and differences among the healthy and infected blueberry samples.

**Figure 3:**
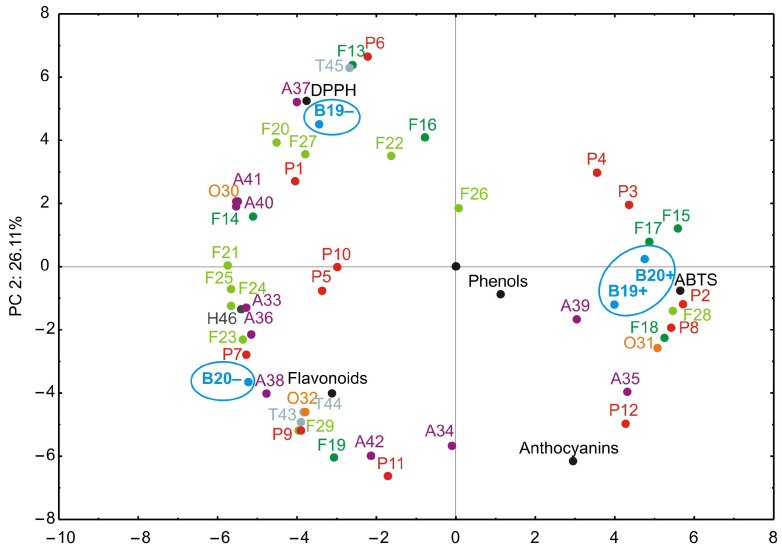
Segregation of four blueberry samples based on detected compounds (42 phenolic compounds, three terpenoids, and one plant hormone, total phenols, flavonoids, anthocyanins, DPPH, and ABTS) determined by principal component analysis (PCA). Variable loadings and sample scores are shown together. The first letter abbreviates the class of compound (P—phenolic acids and derivatives; F (dark)—flavonol aglycones; F (light)—flavonol glycosides and acyl derivatives: O—other phenolic compounds; A—anthocyanins; T—terpenoids; H—plant hormone), while the number corresponds to the specific compound listed in Table 4.

The PCA results are presented in Figure 3. The PCA biplot illustrates the relationships among observed compounds and different blueberry samples. It was found that the first two principal components accounted for 79.32% of the total variance (53.21% and 26.11%, respectively) in the observed parameters.

According to the PCA results, ABTS, caffeic acid hexoside, dimethyl-digallate, caffeoylquinic acid is. I, tetramethyl-digallate, isorhamnetin, patuletin, laricitrin, quercetin 3-*O*-(6″-*O*-rhamnosyl)hexoside, vanilloloside, and peonidin 3-*O*-(6″-acetyl)hexoside, which contributed 3.27%, 3.78%, 2.17%, 1.46%, 3.38%, 3.59%, 2.74%, 3.17%, 3.42%, 2.93%, and 1.05% of the total variance, respectively, demonstrated a positive impact on PC1. Additionally, DPPH (3.56%), caffeoylquinic acid is. III (7.16%), kaempferol (6.60%), myricetin (2.67%), quercetin 3-*O*-pentoside (2.49%), quercetin 3-*O*-hexoside (1.97%), isorhamnetin 3-*O*-(6″-acetyl)hexoside (2.02%), petunidin 3-*O*-hexoside (4.39%), malvidin 3-*O*-(6″-acetyl)hexoside (5.67%), and pentacyclic terpenoid II (6.59%) positively influenced the calculation of PC2, as presented in Figure 3.

PCA revealed that the primary factor driving variation in the phenolic profiles was the infection status of the plants. The first principal component (PC1), explaining over 55% of the variance, effectively separated virus-infected samples (B19+, B20+) from healthy controls (B19−, B20−), underscoring the profound metabolic impact of BlMaV infection. All BlMaV-infected blueberry samples (B19+, B20+) were grouped on the positive side of PC1, while healthy samples (B19−, B20−) are positioned on the negative side of the same axis (Figure 2). In contrast, the year of harvest (2019, 2020) contributed to a secondary source of variation, indicating that environmental factors linked to harvest year played a lesser, though still detectable, role. Variations between 2019 and 2020 may reflect environmental factors, such as temperature and rainfall (Table 2). However, these changes did not overshadow the biochemical perturbations induced by BlMaV. These detailed chemical/statistical analyses clearly demonstrate that viral infection is a dominant factor affecting the phenolic composition of blueberry fruits, leading to general alterations in key phenolic compounds.

Specifically, phenolic acid derivatives such as methyl gallate (**1**), caffeic acid hexoside (**2**), tetramethyl digallate (**9**), and caffeoylquinic acid dimer (**12**) exhibited significantly altered concentrations in infected samples. For example, the levels of tetramethyl digallate (**9**) and caffeoylquinic acid dimer (**12**) were generally increased in virus-infected plants compared to healthy counterparts. Caffeic acid hexoside (**2**) was not even detected in healthy blueberry samples. On the other hand, level of methyl gallate (**1**) was reduced in infected samples. Among the other compound groups, increased amounts of isorhamnetin (**15**), patuletin (**17**), laricitrin (**18**), quercetin 3-*O*-(6″-*O*-rhamnosyl)hexoside (**28**), vanilloloside (**31**), and malvidin 3-*O*-pentoside (**35**) were detected in infected blueberry samples, whilst quercetin (**14**), myricetin (**16**), myricetin 3-*O*-pentoside (**21**), quercetin 3-*O*-hexoside (**22**), myricetin 3-*O*-hexoside (**23**), laricitrin 3-*O*-hexoside (**24**), quercetin 3-*O*-(6″-acetyl)hexoside (**25**), isorhamnetin 3-*O*-(6″-acetyl)hexoside (**27**), naringenin (**30**), procyanidin B-type dimer (**32**), delphinidin 3-*O*-pentoside (**33**), delhinidin 3-*O*-hexoside (**36**), petunidin 3-*O*-hexoside (**37**), malvidin 3-*O*-hexoside (**38**), delphinidin 3-*O*-(6″-acetyl)hexoside (**40**), petunidin 3-*O*-(6″-acetyl)hexoside (**41**), and abscisic acid (**46**) are dominant in healthy samples. All these alterations could suggest impaired biosynthesis or enhanced degradation under viral stress conditions. It seems that anthocyanin content, a key determinant of blueberry pigmentation, and flavonol derivatives were the most influenced by the viral infection, leading to diminished levels in infected berries. For the remaining 19 compounds, no clear trend was observed.

It is well-known that numerous viruses interact with diverse plant species and modulate their metabolic processes. To cope with the biotic stress imposed by viral infection, plants activate the biosynthesis or degradation of a range of secondary metabolites and phytohormones that play a crucial role in their defense mechanisms. The accumulation of certain phenolic acids derivatives and flavonols in infected ‘Duke’ blueberries suggests activation of the phenylpropanoid pathway as part of the defense response against biotic stress, particularly, through enhanced antioxidant capacity and reinforcement of cell wall structures [49]. Conversely, healthy ‘Duke’ samples contained higher amounts of anthocyanins derivatives, which are physiologically linked to normal fruit ripening, pigmentation, and stress regulation. Thus, the virus appears to redirect metabolic fluxes from pigmentation and developmental regulation towards defensive secondary metabolites, reflecting a trade-off between growth/ripening and defense in plant physiology. These findings are well-correlated with our previous publication, which showed that pomological properties, such as fruit length, width and height, shape, fruit weight, and soluble solids content of blueberry ‘Duke’ fruits were not altered by the influence of BlMaV [9]. In several studies, virus infection has been shown to alter the balance between anthocyanin synthesis and phenolic/flavonol accumulation [50,51].

The PCA score plots revealed a clear separation between BlMaV-infected and healthy blueberry samples, indicating that the infection status was the dominant factor explaining the observed variance in phenolic composition. In contrast, neither harvest year (i.e., environmental variation) resulted in distinct clustering, suggesting that the virus-induced changes outweighed seasonal effects. Along PC1, infected samples consistently grouped together, reflecting a shared pattern of altered accumulation of certain phenolic acids and flavonol derivatives, while reductions in specific anthocyanins contributed to the observed separation. These findings highlight the significant metabolic shift triggered by BlMaV infection and provide further support for the notion that viral stress strongly impacts the phenolic balance in ‘Duke’ blueberries.

## 4. Conclusions

In this study, comprehensive profiling of phenolic compounds in ‘Duke’ blueberries revealed distinct metabolic alterations associated with BlMaV infection. A total of 46 compounds were detected, with several flavonols, phenolic acids, and anthocyanins showing virus-related changes. Color correlation analysis highlighted clear patterns of co-variation among metabolites, while PCA demonstrated a consistent separation of healthy and infected samples, confirming that BlMaV infection was the dominant factor shaping phenolic composition, exceeding the influence of harvest year or environmental conditions. The infection induced profound metabolic perturbations, including the accumulation of certain phenolic acid derivatives and flavonol aglycones, along with the depletion of several anthocyanins and related glycosides, thereby disrupting the balance of secondary metabolism. These findings suggest that, despite its widespread cultivation, the cultivar ‘Duke’ displays measurable sensitivity to BlMaV infection at the biochemical level. Consequently, the observed changes provide valuable biochemical markers for monitoring viral stress in blueberries, with broader implications for fruit quality, nutritional value, and plant defense mechanisms. This robust and untargeted study aims to classify the detected compounds and identify one or more compounds of interest for further investigation, including method validation and quantitative analysis. Future studies should further investigate the mechanistic basis of these shifts and their potential impact on consumer-relevant traits.

## Figures and Tables

**Table 1:** Virus presence indication for ‘Duke’ blueberry fruits collection over two successive harvest years.

Viral Status	Harvest Year	
	2019	2020
BlMaV−	B19−	B20−
BlMaV+	B19+	B20+

BlMaV = B: blueberry mosaic-associated ophiovirus. Signs (+) and (−) stand for BlMaV-infected and BlMaV-free blueberry samples, respectively.

**Table 2:** Mean monthly air temperature and precipitation values recorded during the study period (2019–2020).

Month	Average Air Temperature (°C)		Average Precipitation (mm)	
	2019	2020	2019	2020
April	11.7	10.5	166.8	70.0
May	13.1	14.1	110.2	197.2
June	20.4	17.7	328.4	231.0
July	20.1	19.4	107.2	17.4
August	21.3	20.6	71.2	46.6
September	16.5	17.2	2.4	49.6
October	12.4	11.8	5.4	152.6

**Table 3:** Changes in total anthocyanin, flavonoid, and phenolic contents, and antioxidant capacity (DPPH and ABTS), as influenced by BlMaV, over two consecutive years.

	ANOVA	Blueberry Samples			
		B19−	B19+	B20−	B20+
Anthocyanins (mg/100 g fw)	**	112.06 ± 5.02 ^b^	125.05 ± 7.09 ^ab^	129.57 ± 5.51 ^a^	134.34 ± 2.89 ^a^
Flavonoids (mg/100 g fw)	***	106.73 ± 2.45 ^b^	101.45 ± 1.60 ^c^	112.05 ± 0.52 ^a^	108.68 ± 1.81 ^ab^
Phenols (mg/100 g fw)	ns	325.26 ± 4.16	322.61 ± 6.49	324.64 ± 9.67	328.15 ± 4.49
Antioxidant capacity (DPPH, μmol TE/100 g fw)	ns	77.03 ± 4.23	74.14 ± 3.99	73.34 ± 2.78	70.51 ± 6.28
Antioxidant capacity (ABTS, mmol TE/100 g fw)	ns	2.41 ± 0.09	2.50 ± 0.10	2.42 ± 0.07	2.48 ± 0.07

Values with different letters (a, b, c) in the same row denote statistically significant difference (Tukey’s test, *p* < 0.05) among blueberry fruits of different viral status within both harvest years. Means followed by the same letter(s) are not significantly different. ns, **, ***: not significant or significant at, 0.01, 0.001, respectively.

## Data Availability

Dataset available on request from the authors.

## Supplementary Materials

The following supporting information can be downloaded at https://www.mdpi.com/article/10.3390/antiox14111302/s1. Figure S1. Fragmentation patterns (MS/MS spectra) of identified phenolic acids and derivatives: (**1**) Methyl gallate; (**2**) Caffeic acid hexoside; (**3**) Dimethyl-digallate; (**4**) Caffeoylquinic acid is. I; (**5**) Caffeoylquinic acid is. II (Chlorogenic acid)*; (**6**) Caffeoylquinic acid is. III; (**7**) Caffeoylquinic acid methyl ester; (**8**) Tetramethyl-digallate; (**9**) Diferulic acid; (**10**) Caffeoyl coumaroylquinic acid; (**11**) Dicaffeoylquinic acid; (**12**) Caffeoylquinic acid dimer; (ESI^–^). Figure S2. Fragmentation patterns (MS/MS spectra) of identified flavonol aglycones, glycosides and acyl derivatives: (**13**) Kaempferol*; (**14**) Quercetin*; (**15**) Isorhamnetin*; (**16**) Myricetin*; (**17**) Patuletin; (**18**) Laricitrin; (**19**) Syringetin; (**20**) Quercetin 3-*O*-pentoside; (**21**) Myricetin 3-*O*-pentoside; (**22**) Quercetin 3-*O*-hexoside; (**23**) Myricetin 3-*O-*hexoside; (**24**) Laricitrin 3-*O*-hexoside; (**25**) Quercetin 3-*O*-(6″-acetyl)hexoside; (**26**) Syringetin 3-*O*-hexoside; (**27**) Isorhamnetin 3-*O*-(6″-acetyl)hexoside; (**28**) Quercetin 3-*O*-(6″-*O*-rhamnosyl)hexoside (like Rutin)*; (**29**) Isorhamnetin 3-*O*-(6″-*O*-rhamnosyl)hexoside (like Narcissin); (ESI^–^). Figure S3. Fragmentation patterns (MS/MS spectra) of identified other phenolics, pentacyclic terpenoids, and abscisic acid: (**30**) Naringenin*; (**31**) Vanilloloside; (**32**) Procyanidin B-type dimer (like Procyanidin B2)*; (**43**) Pentacyclic terpenoid (like Maslinic or Pomolic acid); (**44**) Pentacyclic terpenoid I (like Arjunolic, Euscaphic or Rotundic acid); (**45**) Pentacyclic terpenoid II (like Arjunolic, Euscaphic or Rotundic acid); (**46**) Abscisic acid; (ESI^–^). Figure S4. Fragmentation patterns (MS/MS spectra) of identified anthocyanins: (**33**) Delphinidin 3-*O*-pentoside; (**34**) Petunidin 3-*O*-pentoside, (**35**) Malvidin 3-*O*-pentoside; (**36**) Delphinidin 3-*O*-hexoside; (**37**) Petunidin 3-*O*-hexoside; (**38**) Malvidin 3-*O*-hexoside; (**39**) Peonidin 3-*O*-(6″-acetyl)hexoside; (**40**) Delphinidin 3-*O*-(6″-acetyl)hexoside; (**41**) Petunidin 3-*O*-(6″-acetyl)hexoside; (**42**) Malvidin 3-*O*-(6″-acetyl)hexoside; (ESI^+^). Figure S5. MS-Base peak chromatograms of blueberry extracts: (a,e) B19– blueberry sample; (b,f) B19+ blueberry sample; (c,g) B20– blueberry sample; (d,h) B20+ blueberry sample; in negative ionization mode (a,b,c,d); in positive ionization mode (e,f,g,h); for peak annotation see retention times in Table 4. Table S1. Peak areas and relative changes of detected compounds in BlMaV-infected and healthy blueberry samples, using UHPLC Q-ToF MS. Comparison of peak areas among samples for each identified compound separately, %.

## Abbreviations

The following abbreviations are used in this manuscript:

BlMaV	Blueberry mosaic-associated ophiovirus
RT-PCR	Reverse transcription polymerase chain reaction
BSV, BlShV	Blueberry shock virus
BLMoV	Blueberry leaf mottle virus
BlScV	Blueberry scorch virus
BSSV	Blueberry shoestring virus
ToRSV	Tomato ringspot virus
TRSV	Tobacco ringspot virus
PRMV	Peach rosette mosaic virus
C3GE	Cyanidin-3-glucoside equivalents
CE	Catechin equivalents
GAE	Gallic acid equivalents
DPPH	2,2-diphenyl-1-picrylhydrazyl
ABTS	2,2′-azino-bis(3-ethylbenzothiazoline-6-sulfonic acid)
UHPLC Q-ToF MS	Ultra-high-performance liquid chromatography-quadrupole time-of-flight mass spectrometry
PCA	Principal component analysis

## References

[1] Ma L., Sun Z., Zeng Y., Luo M., Yang J. (2018). Molecular mechanism and health role of functional ingredients in blueberry for chronic disease in human beings. Int. J. Mol. Sci..

[2] (2025). FAOSTAT. https://www.fao.org/faostat/en/#data/QCL.

[3] Leposavić A., Jevremović D. (2020). Borovnica—Tehnologije Gajenja, Zaštite i Prerade.

[4] Retamales J.B., Hancock J.F. (2018). Blueberries.

[5] Saad N., Olmstead J.W., Jones J.B., Varsani A., Harmon P.F. (2021). Known and new emerging viruses infecting blueberry. Plants.

[6] Thekke-Veetil T., Ho T., Keller K.E., Martin R.R., Tzanetakis I.E. (2014). A new ophiovirus is associated with blueberry mosaic disease. Virus Res..

[7] Ramsdell D.C., Stretch A.W., Converse R.H. (1987). Blueberry mosaic. Virus Diseases of Small Fruits.

[8] Jevremović D., Vasilijević B., Leposavić A., Katanić V. Molecular Characterization of Blueberry Mosaic-Associated Virus in Highbush Blueberries in Serbia. Proceedings of the 6th International Scientific Conference: Modern Trends in Agricultural Production, Rural Development, Agroeconomy, Cooperatives and Environmental Protection.

[9] Jevremović D., Paunović A.S., Leposavić A. (2020). Influence of blueberry mosaic associated virus on some fruit traits of highbush blueberry ‘Duke’. J. Mt. Agric. Balk..

[10] Li R., Mock R., Huang Q., Abad J., Hartung J., Kinard G. (2008). A reliable and inexpensive method of nucleic acid extraction for the PCR-based detection of diverse plant pathogens. J. Virol. Methods.

[11] Jevremović D., Leposavić A., Paunović S. (2016). Incidence of viruses in highbush blueberry (*Vaccinium corymbosum* L.) in Serbia. Pestic. Fitomed..

[12] Taghavi T., Patel H., Rafie R. (2022). Comparing pH differential and methanol-based methods for anthocyanin assessments of strawberries. Food Sci. Nutr..

[13] Ionescu C., Samide A., Tigae C. (2025). Trend in detection of anthocyanins from fresh fruits and the influence of some factors on their stability impacting human health: Kinetic study assisted by UV–Vis spectrophotometry. Antioxidants.

[14] Belew A.A., Hanan G.G.M.W., Meshesha D.S., Akele M.L. (2025). Evaluation of total phenolic, flavonoid contents, antioxidant and antibacterial activity of leaf extracts from *Rhus vulgaris*. Discov. Plants.

[15] Zeb A., Rahman F. (2024). Phenolic profile, total bioactive contents, and antioxidant activity of pear fruits. Food Chem. Adv..

[16] Ding Y., Morozova K., Imperiale S., Angeli L., Asma U., Ferrentino G., Scampicchio M. (2022). HPLC-Triple detector (Coulometric array, diode array and mass spectrometer) for the analysis of antioxidants in officinal plants. LWT-Food Sci. Technol..

[17] Miletić N., Jevremović D., Mitić M., Popović B., Petković M. (2022). Influence of D and Rec strains of plum pox virus on phenolic profile and antioxidant capacity of fresh plum fruits of ‘Čačanska Lepotica’ cultivar. Span. J. Agric. Res..

[18] Vrhovsek U., Masuero D., Palmieri L., Mattivi F. (2012). Identification and quantification of flavonol glycosides in cultivated blueberry cultivars. J. Food Compos. Anal..

[19] Tu P.C., Liang Y.C., Huang G.J., Lin M.K., Kao M.C., Lu T.L., Sung P.J., Kuo Y.H. (2020). Cytotoxic and anti-inflammatory terpenoids from the whole plant of *Vaccinium emarginatum*. Planta Med..

[20] Baenas N., Ruales J., Moreno D.A., Barrio D.A., Stinco C.M., Martínez-Cifuentes G., Meléndez-Martínez A.J., García-Ruiz A. (2020). Characterization of Andean blueberry in bioactive compounds, evaluation of biological properties, and in vitro bioaccessibility. Foods.

[21] Barnes J.S., Nguyen H.P., Shen S., Schug K.A. (2009). General method for extraction of blueberry anthocyanins and identification using high performance liquid chromatography–electrospray ionization-ion trap-time of flight-mass spectrometry. J. Chromatogr. A.

[22] Dragović-Uzelac V., Savić Z., Brala A., Levaj B., Bursać Kovačević D., Biško A. (2010). Evaluation of phenolic content and antioxidant capacity of blueberry cultivars (*Vaccinium corymbosum* L.) grown in the Northwest Croatia. Food Technol. Biotechnol..

[23] Okan O.T., Deniz I., Yayli N., Şat I.G., Öz M., Hatipoglu Serdar G. (2018). Antioxidant activity, sugar content and phenolic profiling of blueberries cultivars: A comprehensive comparison. Not. Bot. Horti. Agrobo..

[24] Shibata Y., Ohara K., Matsumoto K., Hasegawa T., Akimoto M. (2021). Total anthocyanin content, total phenolic content, and antioxidant activity of various blueberry cultivars grown in Togane, Chiba Prefecture, Japan. J. Nutr. Sci. Vitaminol..

[25] Rochat B. (2017). Proposed Confidence Scale and ID Score in the Identification of Known-Unknown Compounds Using High Resolution MS Data. J. Am. Soc. Mass Spectrom..

[26] Subbiah V., Zhong B., Nawaz M.A., Barrow C.J., Dunshea F.R., Suleria H.A.R. (2021). Screening of phenolic compounds in Australian grown berries by LC-ESI-QTOF-MS/MS and determination of their antioxidant potential. Antioxidants.

[27] Wang S., Wang B., Dong K., Li J., Li Y., Sun H. (2022). Identification and quantification of anthocyanins of 62 blueberry cultivars via UPLC-MS. Biotechnol. Biotec. Eq..

[28] Bergmann A.R., Siebeneichler T.J., Oliveira Fischer L., Holz Í.R., Rombaldi C.V., Santos Oliveira B.A., Oliveira Fischer D.L., Silva C.S., Helbig E. (2023). Physicochemical characterization, phenolic composition and antioxidant activity of genotypes and commercial cultivars of blueberry fruits. Cienc. Rural.

[29] Araniti F., Baron G., Ferrario G., Pesenti M., Vedova L.D., Prinsi B., Sacchi G.A., Aldini G., Espen L. (2025). Chemical profiling and antioxidant potential of berries from six blueberry genotypes harvested in the Italian Alps in 2020: A comparative biochemical pilot study. Agronomy.

[30] Zhang Q., Zang H., Guo X., Li S., Xin X., Li Y. (2025). A systematic study on composition and antioxidant of 6 varieties of highbush blueberries by 3 soil matrixes in China. Food Chem..

[31] Mikulic-Petkovsek M., Slatnar A., Stampar F., Veberic R. (2012). HPLC–MSn identification and quantification of flavonol glycosides in 28 wild and cultivated berry species. Food Chem..

[32] Chansriniyom C., Nooin R., Nuengchamnong N., Wongwanakul R., Petpiroon N., Srinuanchai W., Chantarasuwan B., Pitchakarn P., Temviriyanukul P., Nuchuchua O. (2021). Tandem mass spectrometry of aqueous extract from *Ficus dubia* sap and its cell-based assessments for use as a skin antioxidant. Sci. Rep..

[33] Ancillotti C., Ciofi L., Rossini D., Chiuminatto U., Stahl-Zeng J., Orlandini S., Furlanetto S., Bubba M.D. (2017). Liquid chromatographic/electrospray ionization quadrupole/time of flight tandem mass spectrometric study of polyphenolic composition of different Vaccinium berry species and their comparative evaluation. Anal. Bioanal. Chem..

[34] Kim J.H., Kwon R.H., Kim S.A., Na H., Cho J.Y., Kim H.W. (2025). Characterization of anthocyanins including acetylated glycosides from highbush blueberry (*Vaccinium corymbosum* L.) cultivated in Korea based on UPLC-DAD-QToF/MS and UPLC-Qtrap-MS/MS. Foods.

[35] Neto C.C., Stoner G.D., Seeram N.P. (2011). Ursolic acid and other pentacyclic triterpenoids: Anticancer activities and occurrence in berries. Berries and Cancer Prevention.

[36] Similie D., Minda D., Bora L., Kroškins V., Lugiņina J., Turks M., Dehelean C.A., Danciu C. (2024). An update on pentacyclic triterpenoids ursolic and oleanolic acids and related derivatives as anticancer candidates. Antioxidants.

[37] Jaakola L. (2023). Phenolic compounds in *Vaccinium* spp.: Diversity, biosynthesis, and molecular regulation. Acta Hortic..

[38] Cocetta G., Rossoni M., Gardana C., Mignani I., Ferrante A., Spinardi A. (2015). Methyl jasmonate affects phenolic metabolism and gene expression in blueberry (*Vaccinium corymbosum*). Physiol. Plant..

[39] Wang C., Zhang M., Wu L., Wang F., Li L., Zhang S., Sun B. (2021). Qualitative and quantitative analysis of phenolic compounds in blueberries and protective effects on hydrogen peroxide-induced cell injury. J. Sep. Sci..

[40] Gavrilova V., Kajdzanoska M., Gjamovski V., Stefova M. (2011). Separation, characterization and quantification of phenolic compounds in blueberries and red and black currants by HPLC–DAD–ESI-MSn. J. Agric. Food Chem..

[41] Wan C., Yuan T., Cirello A.L., Seeram N.P. (2012). Antioxidant and α-glucosidase inhibitory phenolics isolated from highbush blueberry flowers. Food Chem..

[42] Wang L.J., Wu J., Wang H.X., Li S.S., Zheng X.C., Du H., Xu Y.J., Wang L.S. (2015). Composition of phenolic compounds and antioxidant activity in the leaves of blueberry cultivars. J. Funct. Foods.

[43] Häkkinen S., Heinonen M., Kärenlampi S., Mykkänen H., Ruuskanen J., Törrönen R. (1999). Screening of selected flavonoids and phenolic acids in 19 berries. Food Res. Int..

[44] Cvetković M., Kočić M., Dabić Zagorac D., Ćirić I., Natić M., Hajder Đ., Životić A., Fotirić Akšić M. (2022). When is the right moment to pick blueberries? Variation in agronomic and chemical properties of blueberry (*Vaccinium corymbosum*) cultivars at different harvest times. Metabolites.

[45] Sun J., Lin L.Z., Chen P. (2012). Study of the mass spectrometric behaviors of anthocyanins in negative ionization mode and its applications for characterization of anthocyanins and non-anthocyanin polyphenols. Rapid Commun. Mass Spectrom..

[46] Oliva E., Viteritti E., Fanti F., Eugelio F., Pepe A., Palmieri S., Sergi M., Compagnone D. (2021). Targeted and semi-untargeted determination of phenolic compounds in plant matrices by high performance liquid chromatography-tandem mass spectrometry. J. Chromatogr. A..

[47] Oh H.D., Yu D.J., Chung S.W., Chea S., Lee H.J. (2018). Abscisic acid stimulates anthocyanin accumulation in ‘Jersey’ highbush blueberry fruits during ripening. Food Chem..

[48] Petković M., Lukyanov A., Đurović I., Miletić N. (2025). A novel method for analyzing the kinetics of convective/IR bread drying (CIRD) with sensor technology. Appl. Sci..

[49] Ramaroson M.L., Koutouan C., Helesbeux J.J., Le Clerc V., Hamama L., Geoffriau E., Briard M. (2022). Role of phenylpropanoids and flavonoids in plant resistance to pests and diseases. Molecules.

[50] Mishra J., Srivastava R., Trivedi P.K., Verma P.C. (2020). Effect of virus infection on the secondary metabolite production and phytohormone biosynthesis in plants. 3 Biotech.

[51] Abdelkhalek A., Király L., Al-Mansori A.N.A., Younes H.A., Zeid A., Elsharkawy M.M., Behiry S.I. (2022). Defense responses and metabolic changes involving phenylpropanoid pathway and PR genes in squash (*Cucurbita pepo* L.) following Cucumber mosaic virus infection. Plants.

