# Supplementary material for: Influence of Blueberry Mosaic Disease on Polyphenolic Profile and Antioxidant Capacity of Highbush Blueberry ‘Duke’ Fruits
Source: Antioxidants (Basel). 2025 Oct 29;14(11):1302. doi: 10.3390/antiox14111302 (PMC12649459; doi:10.3390/antiox14111302)
Supplement: Supplementary file 1 [file antioxidants-14-01302-s001.zip › antioxidants-3899028-supplementary.pdf]

antioxidants

MDPI

Article

Influence of Blueberry Mosaic Disease on Polyphenolic Profile and Antioxidant Capacity of Highbush Blueberry 'Duke' Fruits

Nemanja Miletić [1,*], Danijel D. Milinčić [2], Mirjana B. Pešić [2], Biljana Lončar [3], Marko Petković [1], Bojana Vasilijević [4], Darko Jevremović [4]

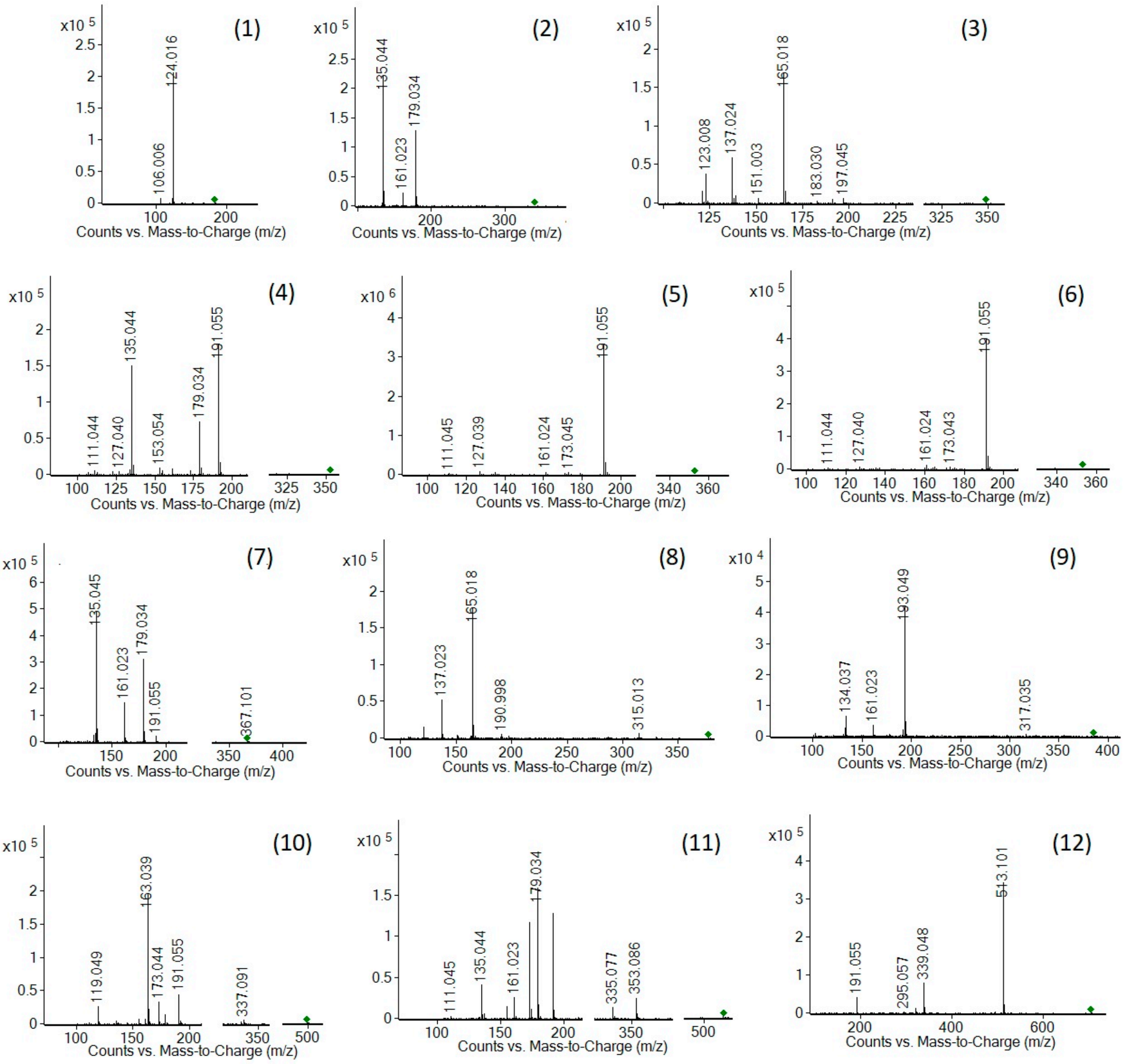

Figure S1. Fragmentation patterns (MS/MS spectra) of identified phenolic acids and derivatives: (**1**) Methyl gallate; (**2**) Caffeic acid hexoside; (**3**) Dimethyl-digallate; (**4**) Caffeoylquinic acid is. I; (**5**) Caffeoylquinic acid is. II (Chlorogenic acid)*; (**6**) Caffeoylquinic acid is. III; (**7**) Caffeoylquinic acid methyl ester; (**8**) Tetramethyl-digallate; (**9**) Diferulic acid; (**10**) Caffeoyl coumaroylquinic acid; (**11**) Dicaffeoylquinic acid; (**12**) Caffeoylquinic acid dimer; (ESI$^-$).

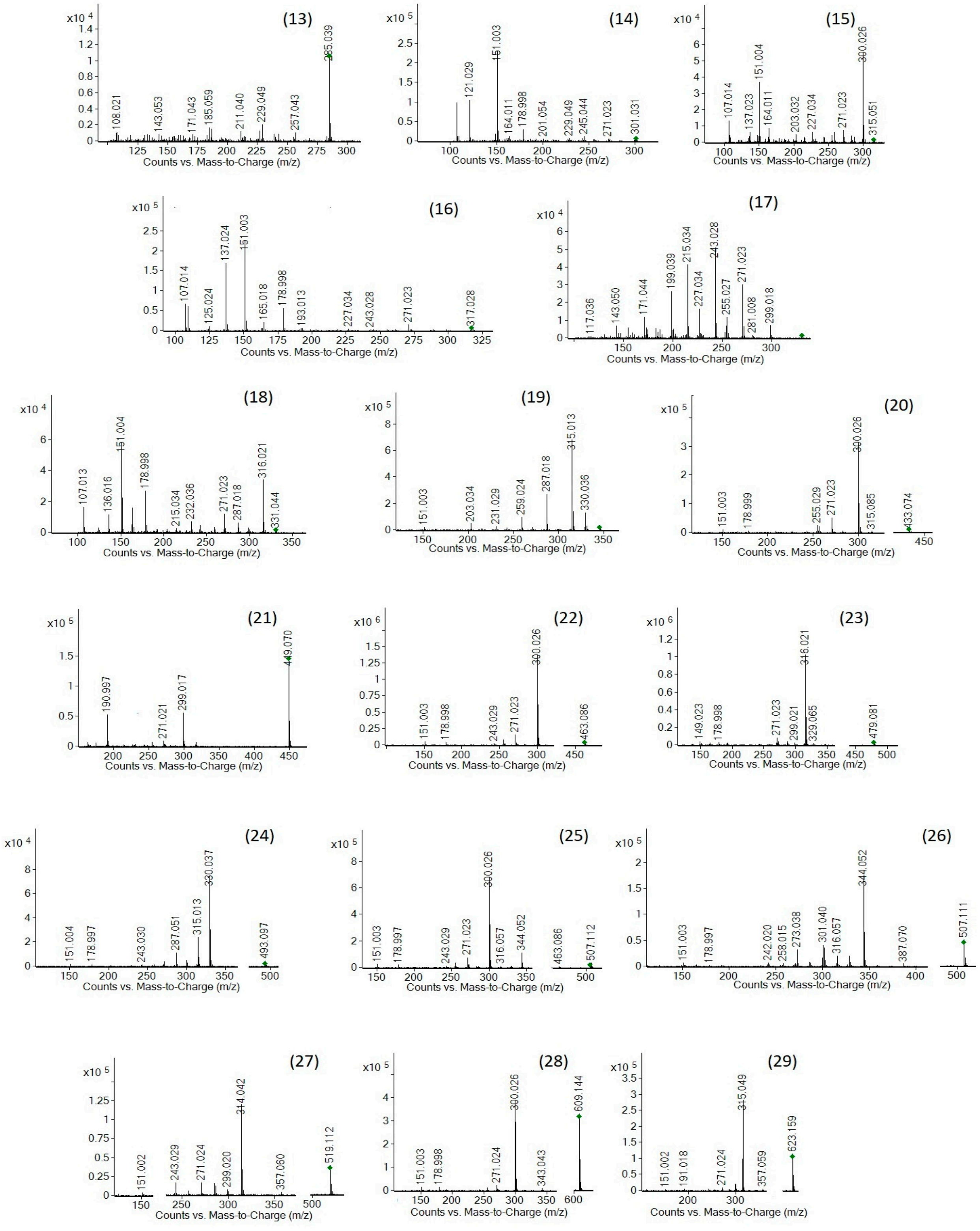

Figure S2. Fragmentation patterns (MS/MS spectra) of identified flavonol aglycones, glycosides and acyl derivatives: (**13**) Kaempferol*; (**14**) Quercetin*; (**15**) Isorhamnetin*; (**16**) Myricetin*; (**17**) Patuletin; (**18**) Laricitrin; (**19**) Syringetin; (**20**) Quercetin 3-*O*-pentoside; (**21**) Myricetin 3-*O*-pentoside; (**22**) Quercetin 3-*O*-hexoside; (**23**) Myricetin 3-*O*-hexoside; (**24**) Laricitrin 3-*O*-hexoside; (**25**) Quercetin 3-*O*-(6''-acetyl)hexoside; (**26**) Syringetin 3-*O*-hexoside; (**27**) Isorhamnetin 3-*O*-(6''-acetyl)hexoside; (**28**) Quercetin 3-*O*-(6''-*O*-rhamnosyl)hexoside (like Rutin)*; (**29**) Isorhamnetin 3-*O*-(6''-*O*-rhamnosyl)hexoside (like Narcissin); (ESI^-).

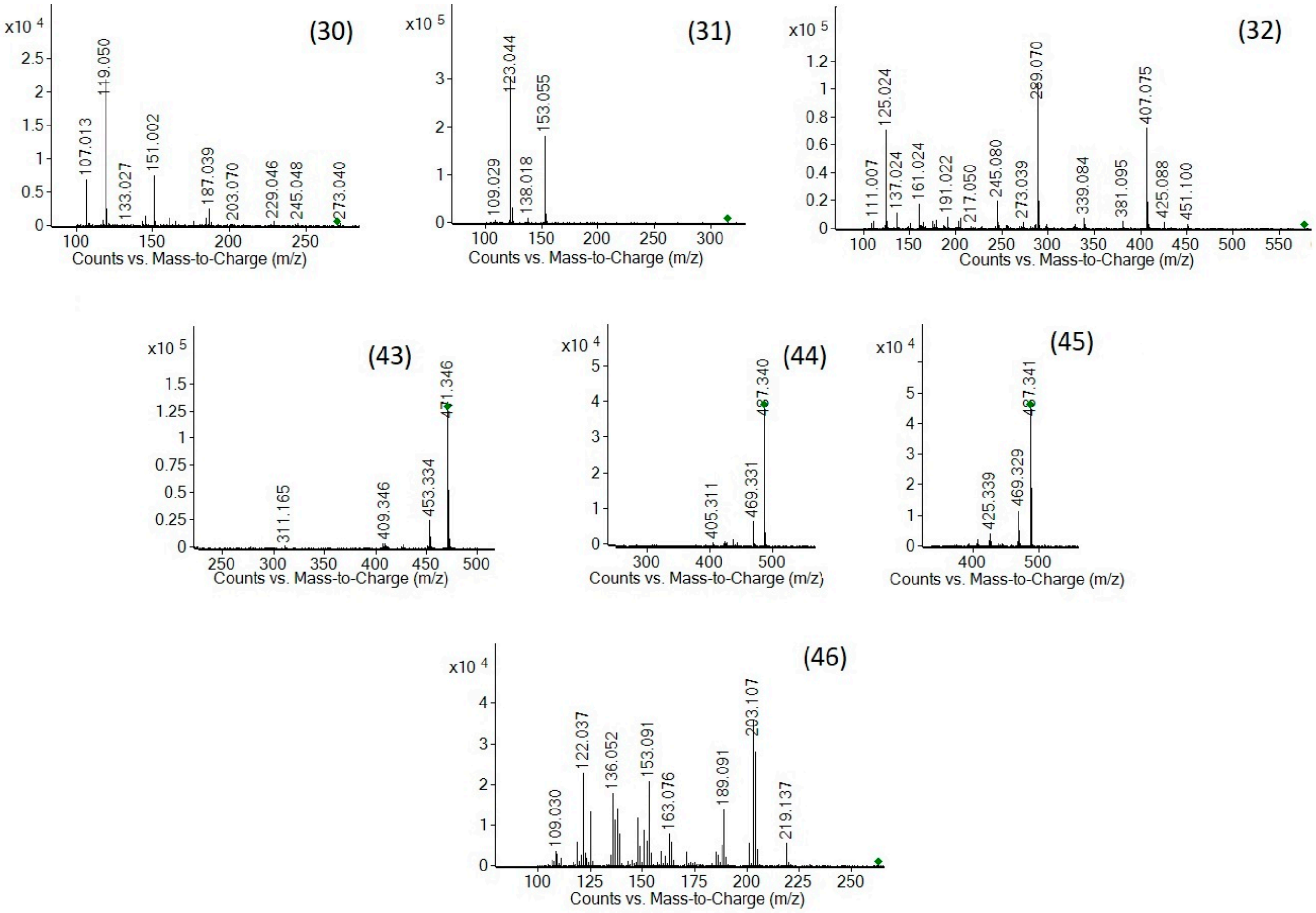

Figure S3. Fragmentation patterns (MS/MS spectra) of identified other phenolics, pentacyclic terpenoids, and abscisic acid: (**30**) Naringenin*; (**31**) Vanilloloside; (**32**) Procyanidin B-type dimer (like Procyanidin B2)*; (**43**) Pentacyclic terpenoid (like Maslinic or Pomolic acid); (**44**) Pentacyclic terpenoid I (like Arjunolic, Euscaphic or Rotundic acid); (**45**) Pentacyclic terpenoid II (like Arjunolic, Euscaphic or Rotundic acid); (**46**) Abscisic acid; (ESI^-).

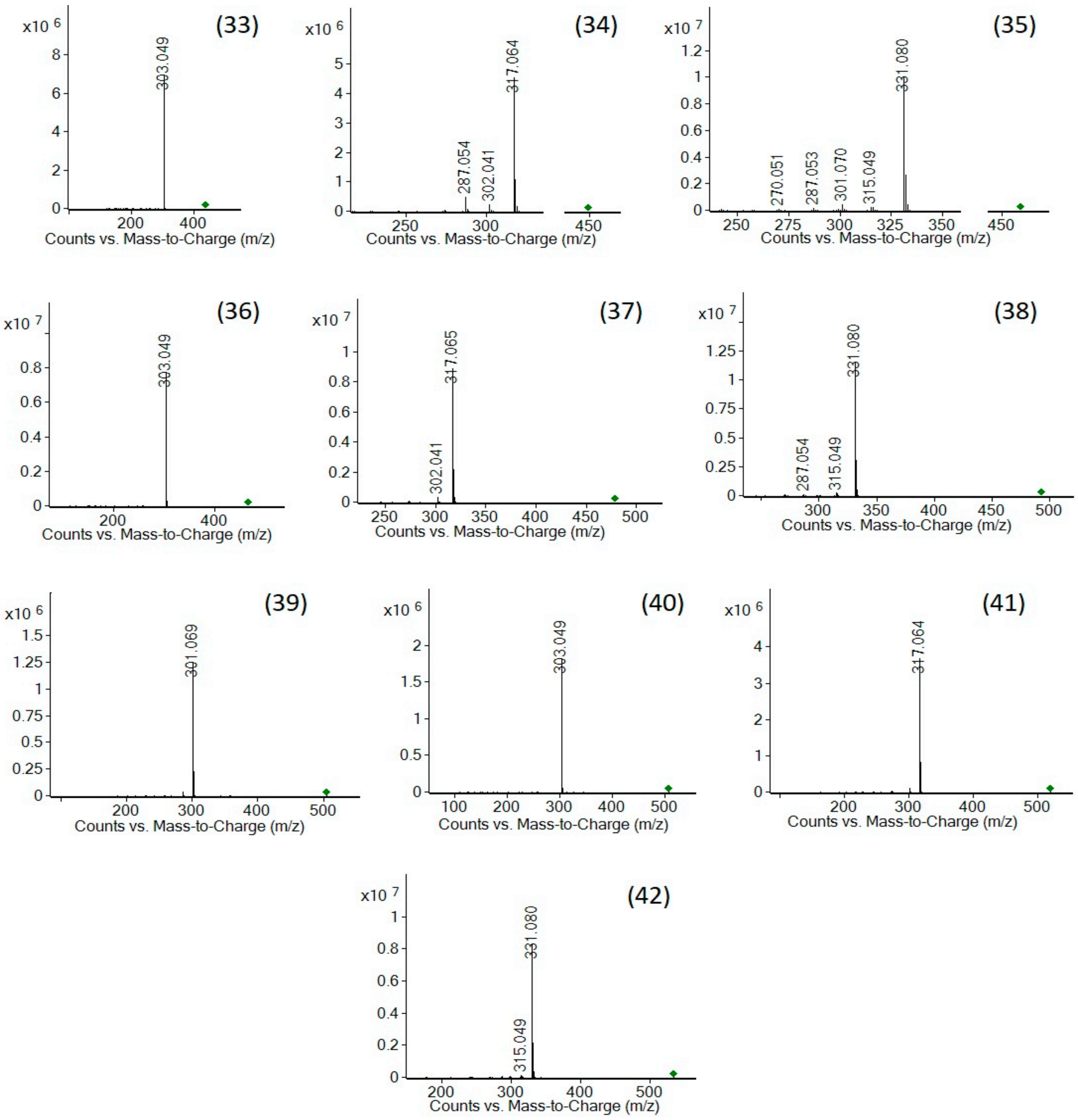

Figure S4. Fragmentation patterns (MS/MS spectra) of identified anthocyanins: (**33**) Delphinidin 3-*O*-pentoside; (**34**) Petunidin 3-*O*-pentoside, (**35**) Malvidin 3-*O*-pentoside; (**36**) Delphinidin 3-*O*-hexoside; (**37**) Petunidin 3-*O*-hexoside; (**38**) Malvidin 3-*O*-hexoside; (**39**) Peonidin 3-*O*-(6''-acetyl)hexoside; (**40**) Delphinidin 3-*O*-(6''-acetyl)hexoside; (**41**) Petunidin 3-*O*-(6''-acetyl)hexoside; (**42**) Malvidin 3-*O*-(6''-acetyl)hexoside; (ESI^+).

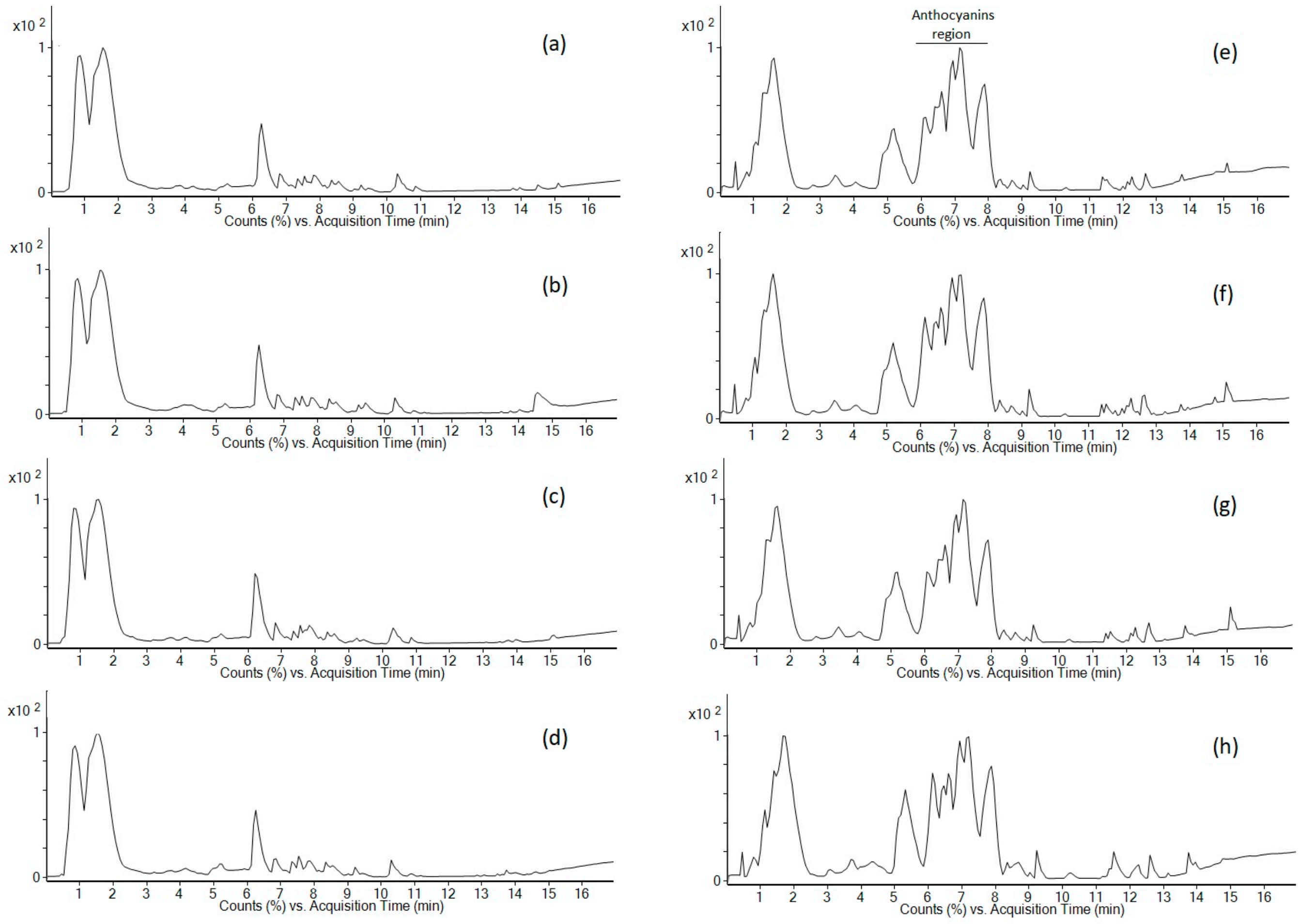

Figure S5. MS-Base peak chromatograms of blueberry extracts: (a,e) B19– blueberry sample; (b,f) B19+ blueberry sample; (c,g) B20– blueberry sample; (d,h) B20+ blueberry sample; in negative ionization mode (a,b,c,d); in positive ionization mode (e,f,g,h); for peak annotation see retention times in Table 4.

Table S1. Peak areas and relative changes of detected compounds in BlMaV-infected and healthy blueberry samples, using UHPLC Q-ToF MS. Comparison of peak areas among samples for each identified compound separately, %.

No.	Compounds name	Blueberry samples			
		B19−	B19+	B20−	B20+
		Phenolic acids and derivatives			
1	Methyl gallate	**19,933,864 (100%)**	7,529,876 (37.8%)	17,884,359 (89.7%)	15,719,831 (87.9%)
2	Caffeic acid hexoside	–	**12,059,295 (100%)**	–	11,421,719 (94.7%)
3	Dimethyl-digallate	10,053,402 (27.7%)	10,066,212 (27.7%)	–	**36,292,948 (100%)**
4	Caffeoylquinic acid is. I	12,756,564 (62.7%)	10,004,872 (49.2%)	7,789,882 (38.3%)	**20,346,291 (100%)**
5	Caffeoylquinic acid is. II	162,253,430 (96.4%)	**168,244,632 (100%)**	166,045,854 (98.7%)	87,498,266 (52.0%)
6	Caffeoylquinic acid is. III	**55,678,569 (100%)**	49,635,113 (89.1%)	50,011,775 (89.8%)	50,985,846 (91.6%)
7	Caffeoylquinic acid methyl ester	38,929,369 (89.5%)	35,951,872 (82.6%)	**43,508,693 (100%)**	35,916,009 (82.5%)
8	Tetramethyl-digallate	6,579,503 (27.7%)	**23,751,406 (100%)**	8,110,654 (34.1%)	18,923,049 (79.7%)
9	Diferulic acid	–	–	**1,511,422 (100%)**	–
10	Caffeoyl coumaroylquinic acid	13,709,121 (98.6%)	13,141,745 (94.5%)	**13,900,223 (100%)**	13,822,357 (99.4%)
11	Dicaffeoylquinic acid	12,809,186 (69.7%)	16,337,472 (88.8%)	**18,388,392 (100%)**	13,527,563 (73.6%)
12	Caffeoylquinic acid dimer	-	**16,249,953 (100%)**	10,614,599 (65.3%)	16,071,515 (98.9%)
		Flavonol aglycones			
13	Kaempferol	**645,959 (100%)**	–	–	–
14	Quercetin	**34,392,125 (100%)**	12,851,611 (37.4%)	30,617,949 (89.0%)	13,493,651 (39.2%)
15	Isorhamnetin	4,880,471 (83.2%)	**5,866,604 (100%)**	4,279,728 (73.0%)	5,629,038 (96.0%)
16	Myricetin	**66,492,918 (100%)**	60,633,933 (91.2%)	32,809,171 (49.3%)	24,666,324 (37.1%)
17	Patuletin	4,348,583 (32.7%)	6,569,222 (49.4%)	2,816,388 (21.2%)	**13,301,873 (100%)**
18	Laricitrin	7,855,752 (78.4%)	**10,025,437 (100%)**	8,152,673 (81.3%)	9,300,202 (92.8%)
19	Syringetin	40,687,867 (43.0%)	51,686,099 (54.7%)	**94,534,714 (100%)**	49,276,518 (52.1%)
		Flavonol glycosides and acyl derivatives			
20	Quercetin 3-*O*-pentoside	**12,834,894 (100%)**	12,325,847 (96.0%)	12,643,442 (98.5%)	12,506,796 (97.4%)
21	Myricetin 3-*O*-pentoside	9,116,589 (80.8%)	–	**11,282,343 (100%)**	–

22	Quercetin 3-*O*-hexoside	**131,784,199 (100%)**	120,791,210 (91.7%)	87,334,539 (66.3%)	62,686,114 (47.6%)
23	Myricetin 3-*O*-hexoside	24,716,015 (52.4%)	4,584,849 (9.7%)	**47,138,116 (100%)**	8,186,795 (17.4%)
24	Laricitrin 3-*O*-hexoside	10,112,893 (77.9%)	5,674,565 (43.7%)	**12,981,317 (100%)**	7,182,979 (55.3%)
25	Quercetin 3-*O*-(6''-acetyl)hexoside	38,441,512 (91.2%)	29,994,186 (71.2%)	**42,130,960 (100%)**	31,405,524 (74.5%)
26	Syringetin 3-*O*-hexoside	16,026,520 (96.2%)	**16,667,170 (100%)**	14,910,711 (89.5%)	14,289,981 (85.7%)
27	Isorhamnetin 3-*O*-(6''-acetyl)hexoside	**6,358,997 (100%)**	5,262,422 (82.8%)	5,264,782 (82.8%)	4,086,750 (64.3%)
28	Quercetin 3-*O*-(6''-*O*-rhamnosyl)hexoside (like Rutin)	20,570,421 (80.6%)	23,767,434 (93.1%)	21,158,654 (82.9%)	**25,532,398 (100%)**
29	Isorhamnetin 3-*O*-(6''-*O*-rhamnosyl)hexoside (like Narcissin)	13,116,681 (91.8%)	13,298,453 (93.0%)	**14,292,924 (100%)**	12,972,946 (90.8%)
	Other phenolic compounds				
30	Naringenin	**2,681,536 (100%)**	2,196,183 (81.9%)	2,588,700 (96.5%)	2,127,862 (79.4%)
31	Vanilloloside	11,348,933 (77.1%)	**14,718,481 (100%)**	11,934,419 (81.1%)	13,408,224 (91.1%)
32	Procyanidin B-type dimer (like Procyanidin B2)	4,921,337 (15.4%)	–	**31,881,468 (100%)**	8,325,285 (26.1%)
	Anthocyanins				
33	Delphinidin 3-*O*-pentoside	192,993,121 (95.9%)	183,882,648 (91.4%)	**201,141,170 (100%)**	169,054,845 (84.0%)
34	Petunidin 3-*O*-pentoside	62,843,646 (37.5%)	**167,536,467 (100%)**	149,374,578 (89.2%)	66,661,714 (39.8%)
35	Malvidin 3-*O*-pentoside	317,017,754 (79.7%)	**397,928,334 (100%)**	346,158,261 (87.0%)	361,598,752 (90.9%)
36	Delhinidin 3-*O*-hexoside	217,427,726 (88.8%)	202,567,003 (82.8%)	**244,781,312 (100%)**	168,381,170 (68.8%)
37	Petunidin 3-*O*-hexoside	**241,159,045 (100%)**	215,057,262 (89.2%)	223,606,229 (92.7%)	217,023,290 (90.0%)
38	Malvidin 3-*O*-hexoside	314,319,064 (92.5%)	311,379,192 (91.6%)	**339,755,467 (100%)**	301,524,134 (88.7%)
39	Peonidin 3-*O*-(6''-acetyl)hexoside	–	**7,022,430 (100%)**	–	–
40	Delphinidin 3-*O*-(6''-acetyl)hexoside	**40,372,321 (100%)**	18,332,483 (45.4%)	33,436,668 (82.8%)	–
41	Petunidin 3-*O*-(6''-acetyl)hexoside	**101,816,179 (100%)**	85,772,142 (84.2%)	98,540,087 (96.8%)	84,460,614 (83.0%)
42	Malvidin 3-*O*-(6''-acetyl)hexoside	255,974,289 (76.8%)	267,999,510 (80.4%)	**333,158,328 (100%)**	290,290,619 (87.1%)
	Other compounds (Terpenoids)				
43	Pentacyclic terpenoid (like Maslinic or Pomolic acid)	–	–	**7,600,865 (100%)**	–
44	Pentacyclic terpenoid I (like Arjunolic, Euscaphic or Rotundic acid)	657,498 (34.9%)	1,066,508 (56.7%)	**1,882,337 (100%)**	–

45	Pentacyclic terpenoid II (like Arjunolic, Euscaphic or Rotundic acid)	**3,365,902 (100%)**	–	–	–
	Other compounds (Plant hormone)				
46	Abscisic acid	19,697,102 (89.7%)	16,131,815 (73.5%)	**21,949,311 (100%)**	15,936,914 (72.6%)

* Highest area is labeled as 100% for each compound. The percentage (%) of each identified compound among blueberry samples were evaluated in relation to the sample with the highest peak area (labeled as 100%), and calculated as a ratio of their areas.
